# Aspergillosis: An Update on Epidemiology, Risk Factors, Diagnosis, Susceptibility, and Treatment

**DOI:** 10.3390/jof12030229

**Published:** 2026-03-21

**Authors:** Carlos Alberto Castro-Fuentes, Juan Pablo Cabrera-Guerrero, Esperanza Duarte-Escalante, Graciela Hernández Silva, Alberto Chinney Herrera, María del Rocío Reyes-Montes

**Affiliations:** 1 Posgrado en Ciencias Biológicas, Facultad de Medicina, Universidad Nacional Autónoma de Mexico (UNAM), Ciudad Universitaria, Avenida Universidad 3000, Coyoacán, Mexico City 04510, Mexico; 2 Departamento de Microbiología y Parasitología, Facultad de Medicina, Universidad Nacional Autónoma de Mexico (UNAM), Ciudad Universitaria, Avenida Universidad 3000, Coyoacán, Mexico City 04510, Mexico; dupe@unam.mx; 3 Unidad de Investigación, Hospital Regional de Alta Especialidad de Ixtapaluca (HRAEI), Servicios de Salud del Instituto Mexicano de Seguro Social para el Bienestar (IMSS-BIENESTAR), Carretera Federal Mexico-Puebla Km 34.5, Ixtapaluca 56530, Mexico; 4 Servicio de Microbiología, Instituto Nacional de Enfermedades Respiratorias “Ismael Cosío Villegas” (INER), Calz. de Tlalpan 4502, Belisario Domínguez Secc 16, Tlalpan, Mexico City 14080, Mexico; jpcguerrero@gmail.com; 5 Servicio de Infectología, Hospital Regional de Alta Especialidad de Ixtapaluca (HRAEI), Servicios de Salud del Instituto Mexicano de Seguro Social para el Bienestar (IMSS-BIENESTAR), Carretera Federal México-Puebla Km 34.5, Ixtapaluca 56530, Mexico; 6 Servicio de Infectología, Instituto Nacional de Enfermedades Respiratorias “Ismael Cosío Villegas” (INER), Calz. de Tlalpan 4502, Belisario Domínguez Secc 16, Tlalpan, Mexico City 14080, Mexico; graciela.hernandez.silva@gmail.com; 7 Centro Nacional de Prevención y Control de Enfermedades (CENAPRECE), Francisco de P. Miranda No. 157, Col. Unidad Lomas de Plateros, Álvaro Obregón, Mexico City 01480, Mexico; a.chinney.h@gmail.com

**Keywords:** aspergillosis, epidemiology, risk factors, diagnosis, susceptibility, treatment

## Abstract

Aspergillosis is one of the most common fungal infections worldwide, caused by various species belonging to the genus *Aspergillus*, affecting both immunocompetent and immunocompromised individuals. The objective of this review was to provide an update on the last five years regarding various aspects of this mycosis, including epidemiology, risk factors, diagnosis, susceptibility, and treatment. The results showed that aspergillosis is distributed throughout the world. Furthermore, *A. terreus* was found to be an increasing causative agent in cases of aspergillosis, along with other less common species. Regarding clinical forms, particularly in the case of Allergic Bronchopulmonary Aspergillosis (ABPA), it is necessary to consider patients with structural lung impairment (Chronic Obstructive Pulmonary Disease (COPD) and Interstitial Lung Diseases). Meanwhile, newly identified risk factors for the development of aspergillosis include chronic obstructive pulmonary disease (odds ratio 1.88) and interstitial lung disease (OR 3.71). Furthermore, the main diagnostic methodologies for aspergillosis were polymerase chain reaction (PCR), matrix-assisted laser desorption/ionization time-of-flight (MALDI-TOF), and next-generation sequencing (NGS). Additionally, the usefulness of isavuconazole compared to voriconazole was demonstrated, representing a better alternative for the treatment of aspergillosis, while novel antifungals such as olorofim and fosmanogepix show excellent results in the management of aspergillosis. Due to the discovery of new risk factors, coupled with antifungal resistance in *Aspergillus* spp. and the wide variety of diagnostic tools, individualized assessment of aspergillosis cases is necessary for the appropriate management of this mycosis.

## 1. Introduction

Aspergillosis is a mycosis caused by species of the genus *Aspergillus*, encompassing a wide variety of clinical forms, including the colonization of pre-existing cavities, allergic and cutaneous forms, otomycosis, and invasive aspergillosis, which affect both immunocompetent and immunocompromised individuals [1]. Currently, 453 species are recognized within the genus *Aspergillus* [2]; of these, *Aspergillus fumigatus*, *A. flavus*, *A. niger*, and *A. terreus* are the primary etiological agents of aspergillosis in humans.

Particularly, *A. fumigatus* is the leading causative agent of aspergillosis worldwide, due—among other factors—to its widespread presence in all types of environments, the size of its conidia, the presence of melanin in its cell wall that protects it from the host’s innate immune response, as well as its ability to produce secondary metabolites that exert immunosuppressive effects on the host [3]. Consequently, the WHO recently included *A. fumigatus* in its list of high-risk priority pathogens [4].

Invasive aspergillosis (IA) represents the clinical form of highest risk for the population with a compromised immune response, and the species most frequently associated with this clinical form are *A. fumigatus*, *A. flavus*, *A. niger*, and *A. terreus*; however, in recent years, several cryptic species have been identified in complicated cases of IA [5]. Among the most important immunosuppression factors associated with IA are individuals with hematological malignancy, prolonged neutropenia, hematopoietic stem cell transplant (HSCT), solid organ transplant (SOT), severe pulmonary disease especially in patients in the intensive care unit (ICU), corticosteroid treatment, and hepatic cirrhosis [6]. Furthermore, predisposition to *Aspergillus* infection has recently been reported in cases of influenza infection (IAPA), SARS-CoV-2 (CAPA), and drug use [6,7].

Currently, the management of aspergillosis remains a challenge due to the non-specificity of diagnostic tools. For example, imaging is suggestive but not specific; additionally, analyses of cell wall components such as 1,3-β-D-glucan (BDG) and Galactomannan (GM), as well as cultures, have methodological limitations [8]. Meanwhile, molecular biology-based tools remain excluded from aspergillosis diagnosis due to a lack of standardization [9]. On the other hand, a recent review by Castro-Fuentes et al. [5] showed that the most widely used methods for the diagnosis of aspergillosis were metagenomic next-generation sequencing (mNGS) and various modalities of Polymerase Chain Reaction (PCR); however, each method has advantages and disadvantages that must be considered.

Likewise, antifungal resistance in *A. fumigatus* and non-*fumigatus* species has led to therapeutic failures, representing another global problem, coupled with the presence of cryptic species with innate resistance [10,11,12].

While review studies on aspergillosis exist, they have all focused on a single clinical form, its diagnosis, susceptibility, and treatment. Therefore, this review aims to comprehensively and concisely summarize all the valuable contributions to date regarding this mycosis. Furthermore, it seeks to establish new ideas aimed at improving the diagnosis and management of aspergillosis.

Considering the above, the objective of this review is to update the current state of the epidemiology, risk factors, diagnosis, susceptibility, and treatment of aspergillosis. A literature review was conducted using major databases such as PubMed and Scopus to evaluate articles published during the last five years (2020–2025). The keywords used included *Aspergillus* AND Aspergillosis AND Epidemiology AND Risk Factors AND Diagnosis AND Susceptibility, AND Treatment. Regarding the selection criteria, specifically for epidemiology, only articles that reported identification methods and the identified *Aspergillus* species were selected. For risk factors, only studies that reported odds ratios and confidence intervals were considered. Finally, for treatment, clinical studies with robust evidence for the management of aspergillosis were included.

## 2. Epidemiology

**Figure 1:**
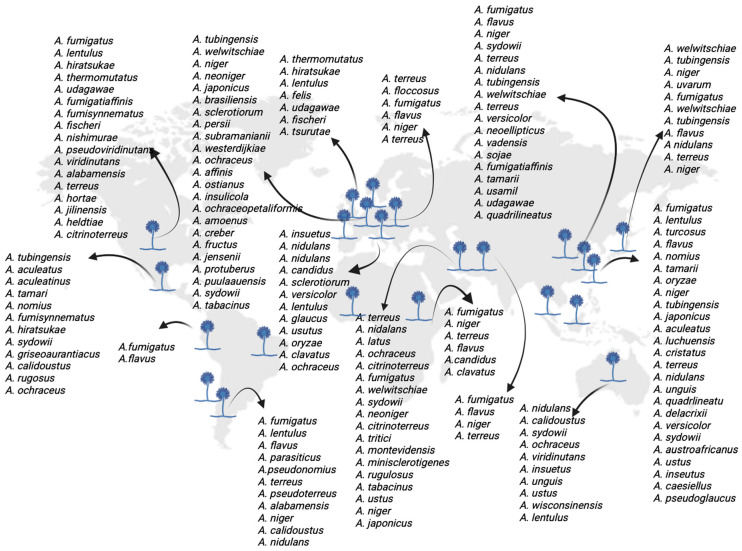
Geographic distribution of species causing aspergillosis across five continents, according to data published in five last years. As can be seen in this figure, *Aspergillus* spp. is widely distributed in different parts of the world, with *A. fumigatus* being the main causative agent of aspergillosis. However, less common species, identified by various methodologies, have been reported in some parts of the world. Therefore, the authors have represented the current distribution of these species, including the most frequent ones. Created in Biorender. Carlos Alberto Castro-Fuentes. (2026) https://www.biorender.com.

The analysis to determine the distribution of species causing aspergillosis worldwide included 67 references from the 2020–2025 period (Table S1) [13,14,15,16,17,18,19,20,21,22,23,24,25,26,27,28,29,30,31,32,33,34,35,36,37,38,39,40,41,42,43,44,45,46,47,48,49,50,51,52,53,54,55,56,57,58,59,60,61,62,63,64,65,66,67,68,69,70,71,72,73,74,75,76,77,78,79], which show data on the *Aspergillus* species identified in these studies, as well as data on geographic origin, sample source, and identification methods. The species identified according to geographic origin showed a distribution across various countries in five continents: (1) America: the United States, Chile, Argentina, Brazil, Mexico, and Peru; (2) Europe: Spain, Switzerland, France, the Netherlands, Italy, Denmark, Austria, and Serbia; (3) Asia: China, Hong Kong, Taiwan, Japan, Korea, Iran, Turkey, Pakistan, Indonesia, Malaysia, and Lebanon; (4) Oceania: Australia; and (5) Africa: Kenya, Ghana and Nigeria (Figure 1). Likewise, regarding the sample origin data, in most of the studies, the isolates were obtained from clinical samples (respiratory tract, external auditory canal, sputum, bronchoalveolar lavage (BAL), wound secretion, tissue, biopsy, pus, etc.), as well as environmental and soil samples. Furthermore, among the most frequently used identification methods, 49 references mention identification through macro- and micromorphological observation of the fungi; 55 studies mention gene sequencing, including β-tubulin (*benA*), calmodulin (*CaM*), and RNA polymerase II subunit B (*RPB2*), as well as Internal Transcribed Spacer (*ITS)* region. Additionally, 14 references include the identification of isolates via Matrix-Assisted Laser Desorption–Ionization Time-of-Flight (MALDI-TOF). Meanwhile, three references utilized Polymerase Chain Reaction-Restriction Fragment Length Polymorphism (PCR-RFLP), microsatellites, and Vitek-2 Compact, respectively, for identification. Other methods, such as ELISA to detect *A. fumigatus* IgG antibodies, lateral flow assay (LFA), and histopathology, were also used in the identification of *Aspergillus*. It is also important to highlight that 40 studies mention the species *A. fumigatus*, in most of them with the highest frequency, alongside other species with a lower frequency (Table S1).

## 3. Clinical Forms of Aspergillosis

Knowledge regarding fungal infections has advanced significantly in recent decades. A notable growth in the number of published articles on aspergillosis has been documented. Between 2019 and 2021, articles on this topic increased from 863 to 1090, representing a 26.3% rise—an increase that reflects the relevance and growing understanding of this topic. Additionally, this increase is attributable to multiple factors, including: the rising prevalence in high-risk populations [80,81,82,83,84], the recognition of new risk groups for Chronic Obstructive Pulmonary Disease (COPD), those in intensive care units, and lung cancer patients [80,85], changes in treatments for high-risk groups such as people living with HIV (PLWHIV) and patients on antifungal prophylaxis [86,87,88], patients previously considered immunocompetent [89,90], climate change [91], as well as the scenario secondary to the COVID-19 health emergency [92,93].

**Figure 2:**
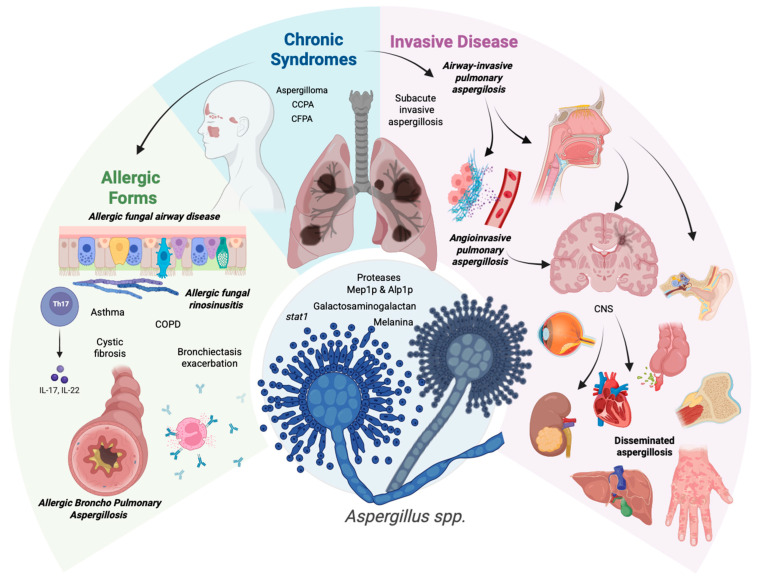
Clinical manifestations of aspergillosis syndromes: allergic syndromes, chronic syndromes, and invasive aspergillosis. The author integrate the main pathogenic mechanisms, triggered by the fungus’s virulence factors (proteases, Mep1p and Alp1p, galactosaminogalactan, *stat 1*, and melanin), involved in the development of the different syndrome complexes associated with *Aspergillus* spp. Regarding allergic forms, the central role of the immune response is highlighted. Furthermore, the clinical continuum that can exist between certain chronic syndromes and invasive disease is emphasized.Created in Biorender. Juan Pablo Cabrera-Guerrero. (2026) https://www.biorender.com.

According to the latest international recommendations [9] which aim to provide a standardized guide for the treatment of aspergillosis based on available evidence, aspergillosis syndromes have been classified into chronic syndromes, allergic syndromes, and invasive aspergillosis (Figure 2). However, these clinical forms should not be viewed as isolated scenarios but as a dynamic spectrum reflecting the interaction between the patient’s immunophenotype, fungal pathogenicity, and the functional anatomy of the affected site [94]. These clinical forms are described below to create an integrative framework that allows for an individualized diagnostic approach to guide and adapt treatment to the patient’s immediate context.

### 3.1. Allergic Forms

#### 3.1.1. Allergic Fungal Rhinosinusitis (AFRS)

Allergic Fungal Rhinosinusitis is a heterogeneous syndrome characterized by chronic paranasal and sinus inflammation with specific features that distinguish it as a subtype of chronic rhinosinusitis. The heterogeneity in its definition is secondary to the characteristics of the populations in which it has been described; however, it can be defined by the presence of type 1 hypersensitivity [95] to certain fungi, bony sinus erosion on computed tomography (CT), and heterogeneous opacities with sinus expansion in imaging studies [96].

Classic diagnostic criteria, according to Bent and Kuhn [97], are divided into major criteria: 1. History of type I hypersensitivity; 2. Nasal polyposis; 3. Characteristic CT findings; 4. Eosinophilic mucin without invasion; 5. Positive fungal stain of sinus contents; and minor criteria: history of asthma, unilateral predominance of disease, radiological imaging showing evidence of bone erosion, positive fungal cultures, serum eosinophilia, and the presence of Charcot-Leyden crystals in surgical specimens. In contrast, the criteria by Deshazo and Swain [98] emphasize the absence of an immunocompromised state and invasive fungal disease.

Regarding the criteria, there is an overlap with other subtypes of chronic sinusitis, which reduces specificity and impacts treatment response. For example, the primary histopathological difference between eosinophilic chronic rhinosinusitis (eCRS) and AFRS is the presence of fungal structures scattered within the eosinophilic mucin [95]. According to Hamilos et al. [99], patients with eCRS present a lower fungal allergic component and lower total IgE but a higher prevalence of asthma, intolerance to NSAIDs/aspirin, and anosmia, as well as higher CT sinus scores and baseline peripheral blood eosinophil counts. This aligns with Nakayama et al. [95], who additionally identified that AFRS patients had an earlier age of onset, greater presence of nasal polyps, as well as more significant tomographic involvement and specific IgE levels for house dust, *Aspergillus* spp., and *Alternaria* spp., with a statistically significant difference (*p* < 0.01). Additionally, the authors categorized AFRS patients into three groups based on their phenotypic characteristics, highlighting the presence of *Aspergillus* spp. specific IgE in all three groups [95].

Although AFRS has classically been etiologically linked to *Aspergillus* spp. due to the induction of inflammation through direct toxicity on the respiratory epithelium and the cleavage of fibrinogen by protease action, recent studies [100]—aimed at characterizing the microenvironment and defining the pathophysiological mechanisms explaining the phenotype and immunotype of AFRS patients (using next-generation molecular techniques)—have shown a greater presence of eosinophilic mucin, type 1 hypersensitivity, and positive fungal cultures/stains compared to patients with non-fungal chronic sinusitis (*p* < 0.1, *p* < 0.02, and *p* < 0.0001, respectively). Nonetheless, it was also observed that chronic sinusitis patients exhibited lower commensal diversity compared to AFRS patients (*p* = 0.008), in whom a higher proportion of species such as *Staphylococcus aureus*, *Streptococcus pneumoniae*, and *Haemophilus influenzae* were identified. These findings suggest a possible pathophysiological association between bacterial species in synergy with fungal hypersensitivity, necessitating a rethink of the pathophysiology and a reconsideration of the role of *Aspergillus* spp., not as an isolated etiological agent, but as a central element within a complex pathophysiological process.

#### 3.1.2. Allergic Bronchopulmonary Aspergillosis (ABPA)

The respiratory epithelium of the airway constitutes the first physical defense barrier against environmental pathogens. However, its role is not limited to preventing the entry of microorganisms; it also plays an essential part in modulating the immune response to allergens and harmful agents, actively participating in the balance between protection and tolerance, primarily through a Th2-type reaction and the release of thymic stromal lymphopoietin, IL-25, and IL-33. Continuously, thousands of *A. fumigatus* conidia and components of other fungi are in close contact with the respiratory epithelium and can trigger an immune response. Furthermore, *Aspergillus* spp. possesses pathogenicity mechanisms such as the production of proteases, specifically elastases, capable of cleaving epithelial tight and adherens junctions. This causes epithelial damage and the release of reactive oxygen species, which potentiate the Th2 response and additionally induce a Th17-type response. In this sense, the term “allergic fungal airway disease” refers to a group of syndromes characterized by evidence of fungal sensitization; the disease spectrum of this term ranges from severe asthma with fungal sensitization to allergic bronchopulmonary aspergillosis (ABPA) [101,102].

ABPA is a complex clinical syndrome characterized by a hypersensitivity reaction to *A. fumigatus*, primarily in patients with difficult-to-treat asthma and cystic fibrosis. Its diagnosis stems from a construct of clinical, immunological, and radiological findings [103]. According to the most recent international consensus, the following criteria are specified: predisposing conditions (asthma, cystic fibrosis, chronic obstructive lung disease, bronchiectasis) or compatible clinico-radiological presentation; essential components (*A. fumigatus*-specific IgE ≥ 0.35 kUA L^−1^, serum total IgE ≥ 500 IU mL^−1^); and other components (positive IgG against *A. fumigatus*, blood eosinophil count ≥ 500 cells µL, thin-section chest computed tomography consistent with bronchiectasis, mucus plugging and high-attenuation mucus, fleeting opacities on chest radiograph). Under this consideration, fungal sensitization is identified as a common denominator with the syndrome of severe asthma with fungal sensitization but without meeting the full diagnostic criteria for ABPA [104].

According to reports by Deka et al. [105] regarding the prevalence and spectrum of fungal sensitization in India, it was observed that up to 50.9% of patients with asthma showed evidence of fungal sensitization. The primary allergen identified was *A. fumigatus* (37.6%), either in isolation (5.5%) or in combination with other fungal allergens (32.1%). Additionally, a high concordance was reported between skin prick tests and serum IgE determination (72.1%; *p* < 0.001). Nonetheless, among patients sensitized to *Aspergillus* spp., it has been documented that up to 37% may develop ABPA [106].

These findings hold clinical relevance, as although not all sensitized patients progress to ABPA, sensitization to *Aspergillus* spp. has also been identified as a risk factor for severe asthma (OR 2.36; 95% CI: 1.29–4.31). Even more significantly, specific sensitization to *A. fumigatus* has shown a stronger association with severe asthma (OR 2.98; 95% CI: 1.32–6.75) compared to sensitization to other species of the *Aspergillus* genus. Furthermore, a higher risk was identified in women and the young population (OR 2.77; 95% CI: 1.16–6.62; OR 2.55; 95% CI: 1.35–4.83) [107].

Recently, Pollock et al. [108] demonstrated that determining the spectrum of lung disease caused by *Aspergillus* spp. has a decisive impact on follow-up outcomes. The authors documented that patients with bronchiectasis of non-cystic fibrosis etiology who presented with *Aspergillus* spp. sensitization, elevated *Aspergillus*-specific IgG, or both conditions, showed a higher frequency of *Pseudomonas aeruginosa* infection (23.9%, 26.4%, and 27.2%, respectively) compared to the control group (17.1%; *p* < 0.0001) [108]. Additionally, these patients showed differences in the frequency of exacerbation, hospitalization, and mortality, identifying elevated *Aspergillus*-specific IgG as a strong predictor of exacerbation and hospitalization (incident rate ratio (IRR), 1.19; 95% CI, 1.05–1.35; *p* = 0.008; IRR, 1.66; 95% CI, 1.37–1.99; *p* < 0.001) [108]. Likewise, the use of inhaled steroids in patients with evidence of sensitization decreased the risk of hospitalization (IRR, 0.70; 95% CI, 0.54–0.90; *p* = 0.006), but this was not the case for patients with specific IgG elevation [108].

Consistent with these findings, Soundappan et al. [89] evidenced that in patients diagnosed with COPD, the presence of *Aspergillus* spp. sensitization was significantly associated with a low percentage of predicted FEV1 (*p* = 0.042) during follow-up.

This scenario allows for a clearer understanding of the clinical relevance of *Aspergillus* spp. sensitization; these findings highlight the importance of identifying an immunological profile within the spectrum of *Aspergillus* lung disease to guide diagnostic, therapeutic, and follow-up approaches.

While specific sensitization to *A. fumigatus* is a benchmark for diagnosis, it is relevant to consider the role of other species. Based on the correlation of serum IgE levels of *A. fumigatus* with other species, Bertlich et al. [109] described that sensitization to *A. fumigatus* does not correlate with evidence of sensitization against other species (*A. versicolor*, *A. niger*, *A. flavus*, and *A. terreus*), highlighting a diagnostic gap in identifying sensitization related to other species of the genus, particularly in patients with atopy.

### 3.2. Chronic Syndromes

#### 3.2.1. Chronic Pulmonary Aspergillosis (CPA)

Chronic Pulmonary Aspergillosis refers to a group of pulmonary diseases defined by the presence of compatible symptoms and tomographic findings with a duration of 3 months or more. It results from the local inflammatory response triggered by the fungus, progressing to cellular damage and fibrosis. Consequently, additional criteria include positive IgG or other evidence of *Aspergillus* spp. infection, as well as the exclusion of differential diagnoses (lung cancer or infarction, vasculitis, or rheumatoid nodules) [110,111]. Within the disease spectrum, the traditionally described forms include simple aspergilloma, chronic cavitary pulmonary aspergillosis (CCPA), chronic fibrosing pulmonary aspergillosis (CFPA), subacute invasive aspergillosis (SAIA), and *Aspergillus* nodule [111,112]. These primarily affect patients with pre-existing structural lung disease, mostly without evidence of severe immunocompromise [94,113].

Diagnostic criteria emphasize the positivity of *Aspergillus*-specific IgG; however, a low diagnostic sensitivity for CPA caused by non-*fumigatus* species has recently been evidenced. This is particularly relevant as these species represent the etiological cause in approximately 25–40% of cases in some regions [114]. Consistent with these data, Davies et al. [115] evidenced that 35.3% of the species identified in CPA patients belonged to *A. flavus*. Similarly, Spruijtenburg et al. [116] reported that up to 56.9% of identified species were *A. fumigatus* and 43.1% were *A. flavus*, attributing this etiological difference to environmental factors, as *A. flavus* proliferates in arid environments while *A. fumigatus* thrives in humid ones. These findings are significant as they were based on isolates from conventional cultures; considering their low diagnostic yield—with a positivity rate of only up to 16.7% [117], they highlight the need to innovate diagnostic strategies and criteria in CPA to overcome current limitations in detecting other *Aspergillus* species.

Additionally, as previously mentioned, CPA patients do not typically present with severe or evident immunocompromise; nevertheless, it has been identified that some patients exhibit mannose-binding lectin deficiency, poor response to encapsulated bacteria vaccines, decreased counts of natural killer cells and T cells, as well as defects in IL-12 and interferon-gamma. All these factors impact the diagnostic yield for identifying *Aspergillus*-specific IgG. Furthermore, Hunter et al. [118], identified a higher diagnostic performance using the LDBio *Aspergillus* IgG/IgM antibody determination compared to the ImmunoCAP *Aspergillus* IgG EIA, with an overall diagnostic sensitivity of 91.6% vs. 74.3% in patients with some form of immunodeficiency.

While diagnostic criteria highlight temporality, this does not necessarily correlate with severity or prognosis across all manifestations of the CPA syndrome. Generally, a mortality risk of approximately 80% is reported, secondary to the development of invasive disease or respiratory failure due to pulmonary fibrosis [117]. Recently, Sengupta et al. [119] reported mortality rates of 15% and 32% at one and five years, respectively.

The term CPA encompasses a syndromic group with shared characteristics, among which certain clinical particularities have recently gained significance due to their impact on prognosis and, consequently, their potential therapeutic implications. Zhong et al. [120] identified in their series that patients with CCPA and SAIA accounted for more than 90% of mortality; however, SAIA patients had a lower survival rate (95.6%, 92.1%, and 92.1% vs. 82.3%, 66.6%, and 51.8% at one, five, and ten years of follow-up, respectively; *p* = 0.001). Among the factors independently related to mortality, they documented a body mass index (BMI) ≤ 20.0 kg/m^2^ (HR 5.311, 95% CI 1.405–20.068, *p* = 0.014), while surgery and the duration of antifungal therapy were associated as survival factors (HR 0.093, 95% CI 0.011–0.814, *p* = 0.032; HR 0.204, 95% CI 0.060–0.696, *p* = 0.011, respectively). Nonetheless, no differences were identified regarding the type of antifungal treatment.

Although SAIA has traditionally been classified among the chronic syndromes, its clinical course (4–12 weeks), the characteristics of reported cases (typically occurring in patients without severe immunocompromise), and its pathophysiological behavior, associated with high mortality, suggest that its diagnosis, treatment, and follow-up should align more closely with the recommendations established for invasive syndromes [112,121].

When characterizing patients with SAIA, a significant difference is observed compared with those with CPA, as a higher proportion of SAIA patients receive systemic glucocorticoid therapy (57.6% vs. 23.3%; OR 2.2, 95% CI 1.3–3.6; *p* = 0.002). This finding reflects the high variability in the reported frequency for this subtype, which is explained by the increase in patients receiving these therapies and, in certain regions, by the decrease in pulmonary tuberculosis cases. In addition, the diagnostic difficulty in distinguishing between sub invasive and invasive forms, especially in the lung transplant population contributes to this variability [122].

HIV infection has been recognized as a risk factor for the development of invasive aspergillosis; however, it has been documented that the initiation of antiretroviral therapy (ART) impacts mortality, decreasing it from 68% to 31% compared to the pre-ART era [123]. Consequently, antiretroviral therapy has shifted the epidemiology over the last 20 years, leading to increased survival and a decrease in opportunistic infections [124]. There are limited evidence and scarce inclusion in consensus guidelines (8% of the population) regarding PLWHIV and chronic pulmonary aspergillosis; a recent study identified that PLWHIV presented a higher proportion of active pulmonary tuberculosis (38% vs. 4.5%, *p* = 0.013). Although no significant difference was demonstrated, a higher proportion of serum GM positivity was identified (43% vs. 13%, *p* = 0.3), as well as in bronchial lavage samples (0.19 vs. 3.57, *p* = 0.2) [125].

Recently, growing evidence has supported the concept that *Aspergillus* lung diseases represent a continuum of syndromic manifestations depending on factors beyond the net immune state. In this regard, Sehgal et al. [126] evaluated the immunophenotype of CPA patients and identified that those with a predominant type 2 response—characterized by a marked elevation in total and specific IgE, as well as *A. fumigatus*-specific IgG and peripheral blood eosinophil counts—presented a lower FEV1:FVC ratio on spirometry. Furthermore, they were more likely to present a fungal ball (88 [74.6%] vs. 145 [62.2%], *p* = 0.023) and had a longer time prior to a disease recurrence episode (11.5 [7.3–27.4] vs. 4 [1.1–8.9] months, *p* = 0.005).

The utility of determining *A. fumigatus*-specific antibodies extends beyond diagnosis and can be considered a non-invasive tool within the continuum of *Aspergillus* lung diseases to differentiate colonization from CPA and invasive disease. Under this premise, Zhang et al. [127] documented, through the evaluation of tomographic findings and the quantitative determination of specific antibodies, that CPA patients presented significantly higher levels of *Aspergillus*-specific IgG compared to invasive aspergillosis (224.44 vs. 150.07 IU/mL, *p* < 0.001) [126]. In summary, these findings highlight an essential strategy for clinical management and follow-up through individualized diagnostic approaches.

#### 3.2.2. Invasive Disease (Invasive Aspergillosis)

Undoubtedly, the invasive aspergillosis syndrome is the topic that has received the most focus and depth, both due to its annual incidence in certain risk populations and its direct attributable mortality (~40–~80%), as well as its mortality rate despite treatment (43–72%) [80]. In this regard, criteria have been developed to guide and facilitate diagnosis and, consequently, direct treatment in patients with diagnostic suspicion [128,129,130,131].

Taking pathological findings as the gold standard for the diagnosis of proven invasive aspergillosis, post-mortem analyses evidenced that only 27% of IA cases were clinically diagnosed. No significant differences were documented regarding risk factors, immunocompromise status, lung disease, or radiographic findings; however, a history of bronchoscopy showed a statistically significant difference, a fact that emphasizes the importance of a targeted approach when the clinical context highlights diagnostic suspicion. Additionally, the study noted that 97% of patients presented pulmonary involvement [132].

Diagnostic and classification criteria have served to standardize patients and facilitate their inclusion in groups for the study of diagnostic tests and antifungal treatment. However, their universal and widespread use in real-world hospital settings has reported significant differences in diagnostic performance. When evaluating their performance, it has been evidenced that the European Organization for Research and Treatment of Cancer and the Mycoses Study Group Education and Research Consortium (EORTC-MSGERC) criteria show 100% concordance with pathological findings, whereas for the FUNDICU, *Asp*-ICU, and *Asp*-ICU-MB criteria, concordance is variable (53%, 4%, and 26%, respectively) in the populations for which they were initially designed. Nevertheless, 5.9% of patients fall outside any classification, and of these, 86% present proven IA through lung biopsy or necropsy [133]. These results, although limited by the risk of selection bias and a sample size (202 patients) that reduces statistical power, provide evidence that current criteria offer only a partial view of a heterogeneous and complex phenomenon. As in the parable of the blind men and the elephant, each system describes one part of the disease, but none manages to capture it in its entirety. Hence, there is an urgent need to optimize, integrate, and expand diagnostic criteria to better reflect the clinical diversity within the continuum of invasive disease caused by *Aspergillus* spp.

Under this concept, Kurita et al. [134] evaluated the EORTC-MSGERC criteria but modified them to adapt to patients with rheumatic inflammatory autoimmune diseases, identifying an increase in sensitivity (100%). Likewise, they identified that the proposed diagnostic category was significantly associated as a risk factor for mortality (HR 2.017; 95% CI 1.067–3.815, *p* = 0.031). This diagnostic category revealed that when patients did not present defined clinical characteristics, they were not categorized under probable IA criteria; furthermore, they presented a higher proportion of interstitial lung disease [134]. Pulmonary involvement, as previously mentioned, along with clinical characteristics, underscores the importance of functional anatomy in understanding the manifestations of aspergillosis syndromes.

#### 3.2.3. Paranasal Sinuses and Airway

Invasive airway infection by *Aspergillus* spp. represents the primary etiology of fungal airway infections (69%), followed by Mucormycetes (22%). Among the main risk factors are diabetes (47.8%) and hematological diseases (39.8%). Both acute and chronic courses have been described. Clinical manifestations are varied, ranging from asymptomatic disease to symptoms reflecting severity, such as ophthalmoplegia, vision loss, proptosis, and stroke [135].

Invasive disease at the pulmonary and airway levels can be divided into two types, according to the anatomical structures affected:

Angioinvasive pulmonary aspergillosis is characterized by direct invasion of pulmonary vessels by *Aspergillus* spp., resulting in thrombosis, infarction, and pulmonary necrosis. It often manifests as pulmonary nodules or areas of consolidation that may progress to cavitation. Pleural involvement typically presents as pleural effusion [136].

Airway-invasive pulmonary aspergillosis, with fungal affection of the basement membrane of the respiratory tract. It manifests as tracheobronchitis, bronchopneumonia, bronchiolitis, and/or atelectasis [136].

This latter manifestation gains relevance in severe respiratory infections, primarily of viral origin such as influenza and COVID-19, due to their capacity to induce anatomical-immunological changes. These changes create an environment that favors the opportunistic capacity of *Aspergillus* spp.; however, there are distinctive histopathological differences, such as a higher fungal load, deep invasion, and greater angioinvasion capacity in IAPA compared to CAPA [137].

After recognizing the respiratory tract as the primary organ affected and the primary site of colonization and infection and considering evidence from post-mortem studies showing that up to 50% of invasive aspergillosis cases present extrapulmonary involvement [138], it is essential to broaden the perspective toward systemic dissemination, addressing the manifestations of invasive disease in other specific organs.

### 3.3. Invasive Aspergillosis: Organ-Specific

#### 3.3.1. CNS Aspergillosis

Aspergillosis affecting the central nervous system (CNS) has been described with a non-specific and heterogeneous presentation pattern but consistently with high mortality (45.1%). It can be secondary to hematogenous dissemination, invasion by contiguity following a paranasal or auditory infection, or secondary to trauma or neurosurgery. Through the action of aflatoxin and the inhibition of phagocytosis, the opsonization of microconidia is reduced, with subsequent neuroinvasion.

In this regard, according to a systematic review that included 235 patients with proven CNS aspergillosis (EORTC/MSGERC), the most frequently identified species was *A. fumigatus* (70.8%), followed by *A. flavus* (18.6%). Furthermore, patients in whom *A. fumigatus* was identified presented higher mortality (52.5% vs. 19%, *p* < 0.05). Interestingly, patients with *A. flavus* identification were mostly immunocompetent and residents of regions with tropical climates (81% and 76.2%, respectively).

The analysis of the risk factors studied did not document any specific comorbidity or predisposing factor. In this same study, pulmonary disease was identified as the most frequent site of dissemination (88.9%), as well as 12.5% of patients with cardiac involvement due to the presence of endocarditis.

The primarily reported symptoms were headache, focal neurological deficit (cranial involvement of the 3rd, 4th, 5th, and 6th cranial nerves), and fever (49.8%, 44%, and 28%, respectively) [139]. However, manifestations vary significantly regarding the dissemination pattern; altered consciousness is reported more frequently in patients with hematogenous dissemination (64% vs. 36%), whereas cranial nerve palsy and headache are more frequent in patients with infection by contiguity (65% vs. 19% and 65% vs. 24%, respectively) [140].

#### 3.3.2. Eye

Fungal ophthalmitis caused by *Aspergillus* spp. has been described as secondary to both hematogenous dissemination [141] and surgical procedures [142]. In cases of endogenous fungal endophthalmitis involving *Aspergillus* spp., it has been reported as the second leading fungal etiology with a frequency of 26.09% [143]. It primarily affects patients with immunocompromise (chronic lymphocytic leukemia, Erdheim-Chester) or comorbidities (septic thrombophlebitis with mitral valve prosthesis), with fundoscopic examination identifying peripheral chorioretinal lesions. *A. fumigatus* is the main species identified, although in some cases, ocular sample cultures yield negative results [141,142,143]. In cases related to surgical procedures, case series have described associations with cataract surgery and intraocular lens placement, likely linked to air conditioning systems. Unlike patients with endogenous origins, these patients were asymptomatic, and physical examination identified a fungal ball in the anterior chamber; furthermore, all cases achieved microbiological identification via culture, identifying *A. fumigatus*. In contrast to these findings, Das et al. [144] reported *Aspergillus* spp. as the primary fungal etiology of endophthalmitis, a discrepancy secondary to the characteristics of the included patients (87.4% post-cataract surgery, 35.6% traumatic, and 17.5% endogenous endophthalmitis) [144].

#### 3.3.3. Cardiovascular: Endocarditis

*Aspergillus* spp. endocarditis represents the second most common cause of fungal endocarditis (1–2% of all cases of infective endocarditis). Among fungal etiologies, its reported frequency is around 30%, second only to *Candida* spp., which accounts for approximately 49.6% of cases [145]. Distinctive features of this clinical spectrum include its primary occurrence in patients with immunocompromise due to solid organ transplant (19.7%) and patients considered immunocompetent but with prosthetic valves or intracardiac devices (34.4%), mainly involving the mitral and aortic valves (36.1% and 29.5%). The main reported species are *A. fumigatus* (47.5%) and *A. flavus* (24.6%). Fungal endocarditis due to *Aspergillus* spp. carries a high complication rate; it is reported that up to 54% of patients experience an embolic episode, with mortality exceeding 50%, particularly in immunosuppressed patients (59.4% vs. 24.1%, OR = 4.09, 95% CI = 1.26–13.19, *p* = 0.02) [146]. Presentation in patients without these risk factors is exceptionally rare; however, it has increased in patients with native valves, where, in addition to solid organ transplant, the presence of autoimmune/inflammatory diseases (20.8%) and chronic diseases without severe immunosuppression (cirrhosis, COPD, diabetes, 12.5%) have been described. In these patients, there is a predominance of mitral, aortic, and tricuspid involvement (60%, 28.9%, and 11.1%, respectively) [147].

#### 3.3.4. Bone: Osteomyelitis and Arthritis

Bone and joint infections of fungal etiology are infrequently described; however, they stand out due to their implications for prognosis and treatment. *Aspergillus* spp. is described as the leading etiological cause, accounting for 26.5% of cases, followed by *Candida* spp. (20.7%). It is primarily reported in patients with a surgical history (26.9%), diabetes (24.3%), or secondary to disseminated fungal infection (21.8%). It mainly compromises the bones of the basicranium, splanchnocranium, other cranial bones, and extremities. The identification of *Aspergillus* spp. is significantly associated with higher mortality (OR 2.7, 95% CI 1.5–4.8, *p* < 0.001) [148].

#### 3.3.5. Gastrointestinal

Gastrointestinal aspergillosis is described as a rare manifestation with high mortality (68.4%); an incidence of 2% has been reported, primarily as a complication of disseminated disease. However, post-mortem studies highlight that this figure may be underestimated, reporting an incidence of up to 17%, with only 0.7% of cases reported as primary infection. Absolutely all reported cases presented with immunocompromise, mainly hematological malignancies (65.9%), solid tumors (7.4%), or solid organ transplant (4.3%). The underdiagnosis is likely explained by a non-specific clinical presentation, with symptoms that can mimic enterocolitis, coupled with non-specific radiological findings [149].

#### 3.3.6. Renal

Invasion of the urinary tract, and primarily the kidneys (81.3%), by *Aspergillus* spp. has been reported with an incidence of 15% in post-mortem studies. Unlike other manifestations of invasive disease, up to 21% of diagnosed patients did not present any comorbidity or immunosuppressive condition. Nevertheless, risk factors identified include diabetes (29.7%), HIV (12.1%), hematological malignancies (11%), and cirrhosis (8.8%). Regarding clinical manifestations, the absence of symptoms in 11% of patients stands out; however, documented findings include flank pain (36.3%), fever (33%), irritative urinary tract symptoms (20.9%), hematuria (15.4%), and macroscopic fungal evidence in the urinary sediment (fungal ball) in 15.4% [138].

#### 3.3.7. Hepatic

Fungal infection affecting the liver and bile ducts is uncommon, with *Candida* spp. being the most frequent causative agent. Hepatobiliary aspergillosis is even rarer and has only been documented in isolated case reports. These include previously healthy patients with a history of biliary surgery, as well as individuals with hematological malignancies, cholangiocarcinoma, or liver transplant recipients [150,151,152,153,154].

#### 3.3.8. Skin and Appendages

Infection of the skin and appendages by *Aspergillus* spp. is also described as a rare manifestation. Although it infrequently causes invasive disease in deep tissues, it has been reported as a manifestation of primary or disseminated disease, mainly in severely immunocompromised or critically ill patients [155].

Three categorical patterns of manifestation are recognized: otomycosis, onychomycosis, and cutaneous aspergillosis. The route of infection is generally secondary to post-traumatic inoculation, surgery, or superinfection following a bacterial infection [156]. Among the primarily affected populations, people living with HIV, burn patients, neonates, recipients of solid organ or hematopoietic stem cell transplants, and patients with a history of trauma or tattoos have been described [157,158]. Clinically, it may manifest as a non-healing skin ulcer or painful necrotic nodules [156].

Finally, a relevant aspect in the development of invasive aspergillosis lies in the intrinsic characteristics of the pathogen; genes acting as potential markers of invasive disease have been identified, such as the *stat1* gene in *A. fumigatus*. It has been demonstrated that infection by mutant species in nematode and murine models results in higher survival rates, as well as a lower pulmonary fungal load (*p* < 0.05; *p* < 0.01, respectively) [159]. These findings acquire clinical relevance when describing the epidemiological characteristics of isolates from patients with IA, in which an in-hospital mortality rate of 35.2% was reported for cases identifying *A. fumigatus*, 10% for *A. niger*, and 50% for *A. tamarii*. Additionally, patients in whom *A. fumigatus* was identified more frequently presented comorbidities such as COPD (11.15%), coronary vascular disease (22.2%), hypertension (31.5%), and diabetes (16.7%), compared to those with *A. niger*, who presented only hypertension (50%) and autoimmune disease (10%), and *A. tubingensis*, who presented COPD (11.1%), hypertension (22.2%), and cancer (22.2%). Likewise, it was described that nearly 70% of the *A. niger* complex isolates met the criteria for pulmonary infection [78].

## 4. Risk Factors for Aspergillosis

The risk of developing aspergillosis depends on various intrinsic and extrinsic host factors; however, while neutropenia was traditionally accepted as the primary risk factor for developing aspergillosis, the role of non-traditional factors that appear to play a significant role in the development of this mycosis has now been evidenced (Table 1).

### 4.1. Intrinsic and Extrinsic Factors of the Adult Host

#### 4.1.1. Hematological Factors

Fungal infection by *Aspergillus* spp. represents one of the primary complications in patients undergoing hematopoietic stem cell transplantation. In this regard, a meta-analysis analyzing 51 studies with a total of 109,155 patients allowed for the identification of risk factors for the development of aspergillosis in this population. These identified factors include prolonged neutropenia, intensified therapy for graft-versus-host disease (GVHD), previous transplantation, use of anti-thymocyte globulin, haploidentical transplantation, high-dose glucocorticoids, Epstein–Barr virus infection, cytomegalovirus (CMV) infection or reactivation (OR 2.57; 95% CI, 0.76–8.73), and low albumin concentration as risk factors for the development of aspergillosis [82].

#### 4.1.2. Oncological Factors

In a study conducted by Zhang et al. [160], the authors retrospectively analyzed 101 patients with lung cancer and a diagnosis of invasive pulmonary aspergillosis. Among the factors associated with in-hospital mortality, low albumin levels (OR 0.80, 95% CI 0.279–1.881, *p* = 0.0025), respiratory failure (OR 12.7, 95% CI 10.2–15.2, *p* = 0.0055), and febrile neutropenia (OR 7.33, 95% CI 5.21–9.45, *p* = 0.0079) were recognized as independent risk factors. Meanwhile, in the multivariate analysis, response to antifungal treatment and respiratory failure were associated with a low odds ratio for successful treatment response (OR 13.3, 95% CI 9.64–16.92, *p* = 0.0447). Likewise, in another study conducted by Teng et al. [161], the authors identified male sex (OR 1.96; *p* = 0.008), past or current smoking history (OR 2.92; *p* < 0.001), chronic obstructive pulmonary disease (OR 1.88; *p* = 0.011), interstitial lung disease (OR 3.71; *p* < 0.001), pulmonary tuberculosis (OR 2.79; *p* = 0.028), and treatment with double lobectomy (OR 2.74; *p* < 0.001) as other risk factors for the development of aspergillosis in this population.

#### 4.1.3. Solid Organ Transplant

In solid organ transplant recipients, risk factors for the development of aspergillosis were evaluated by Pennington et al. [162], who demonstrated that post-transplant renal replacement therapy (OR 3.36; 95% CI, 1.78–6.34) and post-transplant cytomegalovirus disease (OR 2.81; 95% CI, 1.47–5.36) increased the odds of early post-transplant IA. The presence of chronic lung diseases (OR 7.26; 95% CI, 1.05–50.069) and diabetic nephropathy (OR 1.65; 95% CI = 1.10–2.48) have also been evidenced as primary risk factors for the development of IA in renal transplant patients. Likewise, populations requiring post-transplant hemodialysis (OR 3.69; 95% CI = 2.13–6.37) and surgical reintervention (OR 6.28; 95% CI = 1.67–23.66) were at high risk for developing IA. Additionally, a positive association was identified between IA and post-transplant bacterial infection (OR 7.51; 95% CI = 4.37–12.91), viral respiratory tract infection (OR 7.75; 95% CI = 1.60–37.57), cytomegalovirus infection or disease (OR 2.67; 95% CI = 1.12–6.329), and acute graft rejection (OR 3.01; 95% CI = 1.78–5.09) [83]. Another risk factor identified in this population is delayed graft function; as demonstrated in a study of 1963 solid organ transplant recipients, this factor is associated with the development of IA (OR 10.60, 95% CI 1.05–106.84, *p* = 0.045) [163].

On the other hand, specifically in the case of liver transplantation, the use of systemic antibiotics (OR 4.74; *p* = 0.03) and a history of pneumonia (OR 48.7; *p* = 0.01) were identified as risk factors associated with the occurrence of IA [164].

#### 4.1.4. Other Pathologies

Recently, the prevalence of interstitial lung disease (ILD) has increased, and with it, invasive pulmonary aspergillosis (IPA), resulting in high mortality among these patients. Liu et al. [165] conducted a study on 353 hospitalized patients with ILD, divided into a study group (with IPA) and a control group (without IPA). Among the primary risk factors identified in the study group, lymphopenia (OR 2.745, 95% CI 1.344–5.607) and honeycombing (OR 2.915, 95% CI 1.429–5.949) stood out as two risk factors for developing IPA (*p* < 0.05).

Liu et al. [166] identified the clinical characteristics of non-neutropenic patients with IPA. In their study of 151 patients, regression analysis showed that glucocorticoid use (OR 30.223, 95% CI 2.676–341.306), admission to the intensive care unit (OR 0.133, 95% CI 0.023–0.758), and the arterial partial pressure of oxygen/fraction of inspired oxygen (PaO2/FiO2) ratio (OR 0.994; 95% CI: 0.990–0.999) were factors that determined mortality in these IPA patients.

Meanwhile, Qian et al. [167] evaluated the risk factors for exacerbation in patients with ABPA and found that female sex (OR 2.44; 95% CI 1.15–5.16; *p* = 0.020), *A. fumigatus*-specific IgE (OR 1.05; 95% CI 1.02–1.08; *p* = 0.0029), and the presence of bronchiectasis (OR 3.61; 95% CI 1.07–12.21; *p* = 0.039) were independent factors for the development of ABPA exacerbation.

### 4.2. Coinfection

#### 4.2.1. Fungi

In patients coinfected with *Pneumocystis jirovecii* and *Aspergillus* spp., Zhong et al. [168] analyzed the characteristics and risk factors for in-hospital mortality. They reported that lactic acidosis (OR 33.999, 95% CI: 3.112–371.409; *p* = 0.004), a low CD4+ count (<114 cells/µL) (OR 19.343, 95% CI: 1.533–259.380; *p* = 0.022), and high LDH levels (>519 U/L) (OR 11.422, 95% CI: 1.271–102.669; *p* = 0.030) were identified as independent risk factors for mortality in hospitalized patients.

#### 4.2.2. Viruses

In cases of coinfection between influenza viruses and *Aspergillus* spp., Lu et al. [169] analyzed 1720 patients included in observational studies. Solid organ transplant recipients (OR 4.8; 95% CI, 1.7–13.8), hematological malignancy (OR 2.5; 95% CI, 1.5–4.1), immunocompromise (OR 2.2; 95% CI, 1.6–3.1), and those undergoing prolonged corticosteroid treatment prior to hospital admission (OR 2.4; 95% CI, 1.4–4.3) were identified as risk factors for developing IAPA. Additionally, Coste et al. [170] conducted an observational study involving 524 patients with severe RT-PCR-confirmed influenza. Independent risk factors identified included positive *Aspergillus* spp. culture, hepatic cirrhosis (OR 6.7, 2.1–19.4; *p* < 0.01), hematological malignancy (OR 3.3, 95% CI 1.2–8.5; *p* = 0.02), influenza A(H1N1)pdm09 subtype (OR 3.9, 95% CI 1.6–9.1; *p* < 0.01), and vasopressor requirement (OR 4.1, 1.6–12.7; *p* < 0.01). Regarding pneumonia caused by influenza A, a multicenter retrospective study by Zou et al. [171] involving 355 patients found that a smoking history within the last year (OR 6.2, 95% CI 1.7–26) and antibiotic use for more than 7 days prior to admission (OR 4.89, 95% CI 1.0–89) were independently associated with the development of IPA in influenza A (H7N9) patients.

Furthermore, Apostolopoulou et al. [172] analyzed risk factors for invasive pulmonary aspergillosis complicated by non-influenza respiratory viral infections in solid organ transplant recipients. They included 2986 patients who developed infections from respiratory syncytial virus, parainfluenza, and/or adenovirus. The authors identified that a cumulative prednisone dose > 140 mg within the first 7 days (OR 22.6; 95% CI, 4.5–112) and pneumonia at the time of acute non-infectious respiratory infection (OR 7.2; 95% CI, 1.6–31.7) represented the primary risk factors.

In the case of CAPA, the main risk factors were the early use of high-dose corticosteroids for 7 days (>420 mg/week) (OR 1.731; 95% CI, 0.350–8.571) and their prolonged use (2.794; 95% CI, 0.635–13.928). Other studies have highlighted that in patients >60 years, body mass index (OR 1.27, 95% CI 1.08–1.50, *p* = 0.01), solid organ malignancy (OR 5.37, 95% CI 1.35–21.33, *p* = 0.02), and coinfections (OR 5.73, 95% CI 1.40–23.51, *p* = 0.02) are significant risk factors [174]. Similarly, Gangneux et al. [175], in a multicenter study of 565 mechanically ventilated COVID-19 patients, demonstrated through multivariate analysis that age over 62 years (OR 2.34, 95% CI 1.39–3.92, *p* = 0.0013), use of dexamethasone and anti-IL-6 (OR 2.71, 1.12–6.56, *p* = 0.027), and prolonged mechanical ventilation (>14 days) (OR 2.16, 1.14–4.09, *p* = 0.019) were risk factors for CAPA.

Additionally, a meta-analysis by Gioia et al. [92] identified associations with chronic liver disease (OR 2.70, 95% CI 1.21–6.04, *p* = 0.02), hematological malignancy (OR 2.47, 1.27–4.83, *p* = 0.008), chronic obstructive pulmonary disease (OR 2.00, 1.42–2.83, *p* < 0.0001), cerebrovascular disease (OR 1.31, 1.01–1.71, *p* = 0.059), mechanical ventilation (OR 2.83; 95% CI 1.88–4.24; *p* < 0.0001), renal replacement therapy (OR 2.26, 1.76–2.90, *p* < 0.0001), interleukin-6 treatment (OR 2.88, 1.52–5.43, *p* = 0.001), and corticosteroid treatment (OR 1.88, 1.28–2.77, *p* = 0.001). Kim et al. [176] evaluated risk factors in 187 CAPA patients, identifying a dexamethasone dose > 60 mg (OR 3.77; 95% CI, 1.03–13.79) and chronic lung disease (OR 4.20; 95% CI, 1.26–14.02) as independent factors. Furthermore, azithromycin treatment for 3 days or more has been associated with probable IPA in SARS-CoV-2 patients (OR 3.1, 95% CI 1.1–8.5, *p* = 0.02) [177].

Recently, Melenotte et al. [178] conducted a case–control study to identify risk factors for persistent SARS-CoV-2 presence, which increases aspergillosis risk. Fever and lymphocytopenia were associated with viral persistence (OR 3.3, 95% CI 1.01–11.09, *p* = 0.048 and OR 4.3, 95% CI 1.2–14.7, *p* = 0.019, respectively). Unvaccinated patients showed a higher probability of viral persistence and subsequent aspergillosis development (OR 6.6, 95% CI 1.7–25.1, *p* = 0.006).

Another rare coinfection involves the Phlebovirus causing severe fever with thrombocytopenia syndrome (SFTS). Secondary IPA may develop; Hu et al. [179] reported that a CD4+ count < 68 cells/mm^3^ combined with a CD8+ count < 111 cells/mm^3^ (OR 0.218, 95% CI 0.059–0.803, *p* = 0.022) is a significant risk factor. Additionally, IL-6 > 99 pg/mL combined with IL-10 > 111 pg/mL (OR 17.614, 95% CI 2.319–133.769, *p* = 0.006) and a brain natriuretic peptide level > 500 pg/mL were independent risk factors for IPA in SFTS patients.

### 4.3. Hospital Context

Seybold et al. [180] reported risk factors for mortality from aspergillosis in hematological patients with pneumonia in ICU. A positive GM result (OR: 3.1; 95% CI: 1.2–8.0; *p* = 0.021) represented a risk factor for mortality, as well as reactivation of pulmonary cytomegalovirus at the time of intubation (OR: 5.3; 95% CI: 1.1–26.8; *p* = 0.043).

Dabas et al. [181] analyzed clinical and microbiological data, susceptibility, and risk factors to identify determinants of *Aspergillus* spp. infection and 30-day mortality. Among the risk factors for 30-day mortality, the isolation of *A. fumigatus* (OR 1.9, 95% CI 0.8–4.6), *A. nidulans* (OR 1, 95% CI 0.08–12), and *A. niger* (OR 1.2, 95% CI 0.25–5.7) were associated with unfavorable results. Similarly, the administration of liposomal amphotericin B (OR 1.04, 95% CI 0.3–3.55) and the combination of liposomal amphotericin B with posaconazole (OR 3.33, 95% CI 0.28–38.7) were noted. Additionally, age (OR 1.03, 95% CI 1.01–1.05), sex (OR 1.1, 95% CI 0.47–2.56), ICU stay (OR 4.27, 95% CI 1.73–10.53), hematological malignancy (OR 2.48, 95% CI 1.07–5.73), chronic kidney disease (OR 3.67, 95% CI 1.6–8.5), prolonged corticosteroid use (OR 1.56, 95% CI 0.7–3.48), mechanical ventilation (OR 2.77, 95% CI 1.21–6.36), sepsis (OR 3.67, 95% CI 1.15–11.72), and a high galactomannan antigen index value of ≥1 (OR 1.6, 95% CI 0.72–3.56) were identified as statistically significant variables in predicting mortality for these hospital patients with invasive aspergillosis.

In a retrospective study of 140 patients diagnosed with invasive pulmonary aspergillosis admitted to the ICU due to acute kidney injury (AKI), the primary independent risk factors associated with IPA were chronic lung disease (OR 4.122, 95% CI 1.724–9.844, *p* = 0.001), intermittent positive pressure ventilation rate (OR 3.153, 95% CI 1.096–9.076, *p* = 0.033), acute kidney injury (OR 13.364, 95% CI 4.524–39.481, *p* < 0.001), and corticosteroid use within one year (OR 2.890, 95% CI 1.201–6.954, *p* = 0.018) [182].

### 4.4. Pediatrics

Risk factors for other forms of aspergillosis, such as ABPA, have also been analyzed. Celik et al. [183] conducted a study including 259 children with asthma. The identification of *A. fumigatus* was associated with a higher probability of being male (OR 2.45), atopic dermatitis (OR 3.159), sensitivity to another fungal genus (*Alternaria* spp.) (OR 10.37), and a longer duration of asthma (OR 1.266).

On the other hand, it has been evidenced that cystic fibrosis in pediatric patients predisposes to the development of aspergillosis. Breuer et al. [184] evaluated the association between *Aspergillus* spp. infections and lung disease in children with cystic fibrosis. The authors identified that risk factors associated with the presence of *Aspergillus* species include neutrophilic inflammation—specifically IL-8 levels (OR 0.5, 95% CI 0.3–0.7, *p* < 0.001), neutrophil count percentage (OR 8.2, 95% CI 4.8–11.6, *p* < 0.001), neutrophil elastase levels (OR 0.5, 95% CI 0.3–0.7, *p* < 0.001), and the number of admissions for intravenous antibiotic therapy (OR 0.2, 95% CI 0.1–0.3, *p* = 0.008).

## 5. Diagnosis of Aspergillosis

Currently, a wide variety of diagnostic alternatives exist for cases of aspergillosis; however, to date, there is no single method that is useful in all suspected cases. While they have demonstrated diagnostic utility, they also present limitations across different clinical contexts and sample types.

### 5.1. Culture of Sterile Samples

Undoubtedly, culture remains the first-choice diagnostic methodology in aspergillosis cases, particularly for the recognition of macromorphological and micromorphological features that allow for the identification of the etiological agent at the genus level. However, this tool is primarily limited by its low positivity rate from clinical samples of sterile sites, as well as the time required for fungal isolates to grow. Consequently, efforts have been made to evaluate the predictive value of the primary recommended clinical samples that allow for discrimination between colonization and infection, including BAL [9].

### 5.2. BAL

Due to the wide distribution of *Aspergillus* spp. in the environment, the most acceptable approach for diagnosing aspergillosis in patients is the obtaining of clinical samples from the lower respiratory tract, such as BAL, although sputum is considered a second alternative [185]. Regarding the diagnosis of chronic pulmonary aspergillosis—which encompasses a spectrum of diseases caused by *Aspergillus* spp., such as chronic cavitary pulmonary aspergillosis, subacute invasive pulmonary aspergillosis, and chronic necrotizing pulmonary aspergillosis—BAL is the preferred sample type [186].

### 5.3. Sputum

It is important to mention that, although BAL is the most widely recommended clinical sample in cases of suspected aspergillosis, it often cannot be obtained. Therefore, an alternative is sputum; in this regard, it is important to bear in mind that its presence does not necessarily indicate infection. However, according to Dobias et al. [8], this sample has quite acceptable positive predictive values for patients with acute leukemia (100%), neutropenia (94%), and bone marrow transplant (82%), while presenting the lowest positive predictive values for the HIV population (14%) and solid cancer (0%). Particularly in the chronic clinical form of aspergillosis, it is known that only 50% of sputum samples from these patients result in a positive fungal culture, although this yield can be improved by using multiple samples and increasing the volume [186].

### 5.4. Serum

Regarding serum samples, they are generally considered when the clinical form of aspergillosis has progressed to its invasive form, which is the clinical form with the highest mortality rate across different vulnerable patient groups [80]. Thus, serum is a very useful sample for diagnosing invasive aspergillosis through the quantification of *Aspergillus* spp. cell wall constituents, such as galactomannan and 1,3-β-D-glucan.

According to the Infectious Diseases Society of America (IDSA), serum galactomannan is recommended as an accurate marker for IA in adults and pediatric patients, particularly in hematological populations and hematopoietic stem cell transplant patients. While the 1,3-β-D-glucan assay can also be performed using this sample type in the same populations, it is not specific for detecting *Aspergillus* spp. [9].

### 5.5. Non-Culture-Based Methods

Despite the high yield of culture from clinical samples, this methodology alone is insufficient for diagnosis. Currently, in addition to the limitations, the emergence of cryptic species within the genus *Aspergillus* necessitates the implementation of additional tools for species-specific identification, considering the morphological similarities between closely related species [42]. This is particularly critical due to its impact on establishing treatment for aspergillosis, as cryptic species such as *A. lentulus* and *A. tubingensis* have been recognized as causative agents of invasive and chronic pulmonary aspergillosis, respectively [20,187].

### 5.6. Galactomannan and 1,3-β-D-Glucan

Galactomannan is an *Aspergillus* spp. cell wall polysaccharide released into the tissue during fungal growth and can be detected using an immunoassay technique [188]. In specific contexts where a clinical sample for culture cannot be obtained or results are negative, biomarker alternatives such as GM and BDG have been highly useful in diagnosing invasive aspergillosis. Given the widespread availability of GM detection assays worldwide, this has become a diagnostic tool routinely used in cases of suspected aspergillosis.

The EORTC/MSGERC widely recommends the use of the GM biomarker with different cutoff values depending on the sample type: Single serum or plasma: ≥1.0; BAL fluid: ≥1.0; or a single serum or plasma: ≥0.7 with a concurrent BAL fluid result of ≥0.8 [128]. Conversely, for non-neutropenic patients, a GM cutoff value of 0.5 is used to define aspergillosis cases [128]; serum GM tests in these patients are often unreliable, as negative results may stem from early antigen clearance from the bloodstream by neutrophils [189]. Despite the great utility of this biomarker, lack of specificity and false positives—due to the use of antibiotics like piperacillin-tazobactam in its generic formulation—have been demonstrated [190]. Additionally, conditions such as chronic gastrointestinal disease may lead to false positives in GM studies [9]. False-positive results in BAL also include *Aspergillus* spp. colonization without clinical or radiographic evidence of infection, as well as other invasive fungal infections caused by non-*Aspergillus* genera [8].

Regarding chronic pulmonary aspergillosis, the mean Optical Density Index (ODI) value in BAL is 4.5, compared to 0.43 in groups without CPA. In the context of CPA, GM has demonstrated sensitivity and specificity ranging from 68 to 77% and 77 to 93%, respectively. Furthermore, the superiority of BAL over serum GM determination in patients with CPA has been established [8].

Like GM, 1,3-β-D-glucan is a cell wall component considered a pan-fungal biomarker, as it is identified in other medically important fungal genera such as *Candida* spp., *Saccharomyces*, *Fusarium* spp., *Trichosporon* spp., *Acremonium* spp., or *P. jirovecii* [191]. This biomarker is identified in patients with acute pulmonary fungal hypersensitivity. In patients with ABPA, high concentrations of 1,3-β-D-glucan are observed. A positive threshold of ≥80 pg/mL is recommended for invasive pulmonary aspergillosis in both non-neutropenic and neutropenic patient populations [8]. In CPA, the sensitivity and specificity of GM and BDG in BAL were 78% and 90%, and 78% and 73%, respectively [8].

In serum, BDG reports a sensitivity of 81% and a specificity of 61% for the diagnosis of IA. In contrast, for CPA, the sensitivity of serum BDG is very low [192].

### 5.7. Molecular-Based Techniques

#### Polymerase Chain Reaction (PCR)

Polymerase Chain Reaction (PCR) has contributed significantly to the diagnosis of many human infections and has become a routine diagnostic methodology in many laboratories. However, until early 2020, it was not considered among the diagnostic tools for aspergillosis.

Due to efforts focused on standardizing methodology, quality control, and clinical validation by the FPCRI (Fungal PCR Initiative), the revised EORTC-MSGERC guidelines now include positive PCR tests for *Aspergillus* spp. in plasma, serum, or whole blood, as well as BAL [128]. Notably, the utility of PCR is highlighted in immunocompromised patients and those with hematological malignancies who are not receiving prophylactic treatment [110].

Currently, many validated and standardized assays are commercially available. These include *A. fumigatus* Bio-Evolution, artus^®^, AsperGenius^®^ Species and AsperGenius^®^ Resistance, *Aspergillus* spp. ELITe MGB^®^ Kit, AspID, FungiPlex^®^ *Aspergillus* and FungiPlex^®^ *Aspergillus* Azole_R, LightCycler Septifast, Magicplex Sepsis Real-Time Test, MycoReal *Aspergillus*, MycoGENIE^®^ *Aspergillus* Species, and MycoGENIE^®^ *Aspergillus fumigatus* and resistance TR34/L98H [193]. These assays are highly useful as they identify the etiological agent of aspergillosis at the species level and can detect resistance to the antifungals available for treatment.

Recently, a review by Castro-Fuentes et al. [5] showed that PCR assays, in their multiple variants, are among the most widely used molecular methods for the diagnosis of aspergillosis, with BAL, serum, blood, tissue, and sputum being the most used biological samples. Furthermore, they mention that qPCR has been the most utilized method for clinical forms of aspergillosis such as IA, CAPA, rhinosinusitis, chronic pulmonary aspergillosis, and pulmonary aspergillosis. In this regard, the real-time diagnostic capability of this technique is highlighted, primarily by amplifying targets such as 18S and 28S rRNA. However, there is an inversely proportional relationship to the amount of *Aspergillus* DNA present in the patient’s sample. The qPCR technique has demonstrated a sensitivity ranging from 40 to 100% with a specificity of 33–100%. Likewise, multiplex PCR has been highly useful in the diagnosis of IA and CAPA, also implementing 18S and 28S rRNA as targets, and adding the capability to simultaneously detect *A. fumigatus*, *A. flavus*, *A. niger*, and *A. terreus*, with a sensitivity of 47.6–84% and a specificity of 64.7–100%. Meanwhile, nested PCR has enabled the diagnosis of IA, CPA, and rhinosinusitis through the 18S and 28S genes, with a sensitivity of 40–100% and a specificity of 74–100%. Finally, the authors state that end-point PCR, using the *BenA*, *CaM*, and Internal Transcribed Spacer (ITS) genes as well as 18S rRNA, has been primarily used for the diagnosis of IA.

Regarding nucleic acid amplification via PCR, it should be noted that when performed using blood samples, it allows for a screening strategy in transplant patients, neutropenic patients, ICU patients, and in cases of influenza or COVID-19 infection. Conversely, the prophylactic use of antifungals reduces the sensitivity of PCR in BAL [193]. Additionally, efforts focused on the development of multiplex qPCR assays to identify the main etiological agents of aspergillosis (*A. fumigatus*, *A. flavus*, *A. niger*, and *A. terreus*) have yielded results with high sensitivity and specificity in an hour and a half [194]. This promising multiplex qPCR methodology is characterized by simplified analysis and cost reduction; however, its use in biological samples must still be validated.

It is important to mention the specific context of *A. terreus* as an emerging pathogen within *Aspergillus* spp. It is the fourth leading etiological agent of invasive pulmonary aspergillosis, primarily affecting cancer patients, and is notable for its intrinsic resistance to Amphotericin B [195]. Consequently, the utility of PCR has become prominent, allowing for species-specific identification as well as the recognition of resistance patterns [18].

### 5.8. Droplet Digital PCR (ddPCR)

An alternative PCR method for aspergillosis diagnosis is the novel droplet digital PCR (ddPCR), which offers greater sensitivity than qPCR for the diagnosis of bacterial, viral, and fungal infections. Additionally, it demonstrates higher precision and absolute quantification of the target DNA [196]. The basis of this technique is the partitioning of the PCR mixture into segregated droplets that are a thousand times smaller, allowing the amplification of the target of interest within each droplet, which is subsequently quantified by fluorescence. Based on the emitted fluorescence signal, amplification indicates a positive result, whereas the absence of fluorescence yields a negative result [197].

To date, there is only one study describing the use of this method for the clinical diagnosis of invasive pulmonary aspergillosis. Liu et al. [198] evaluated the diagnostic capacity of this technique in 163 patients with suspected IPA. Using BAL and/or plasma, IPA was diagnosed with a sensitivity of 50.8%, compared to culture (44.3%). Nevertheless, a superiority in IPA diagnosis using BAL versus plasma was identified (*p* < 0.01), with a specificity of 94.4%, proving to be a useful tool for IPA diagnosis.

### 5.9. Universal Digital High-Resolution Melt (U-dHRM)

Another recently described technique is universal digital high-resolution melt (U-dHRM). This technique is characterized by its ability to detect a wide variety of pathogens at the genomic level using machine learning algorithms; Training is based on the amplification of specific regions of the fungi by primers such as ITS [5].

Goshia et al. [199] developed a universal fungal assay that generated melting curves as specific signatures for 19 clinically relevant pathogens. Training was conducted using a machine learning algorithm to detect target curves. From 73 BAL samples, accurate identification of 97% of fungal organisms, including *Aspergillus* spp., was achieved in approximately 4 h. Furthermore, the developed methodology allowed for the identification of pathogen mixtures in 19% of cases and showed good sensitivity in the diagnosis of invasive infection.

To date, the speed and capacity of U-dHRM to identify and quantify clinically relevant pathogens in samples with diverse etiological agents, as well as the detection of emerging opportunistic pathogens, contribute to patient improvement by informing therapeutic management decisions.

### 5.10. Nanopore-Targeted Sequencing (NTS)

Zhang et al. [200] developed a targeted sequencing method known as nanopore-targeted sequencing (NTS). This method is characterized by the detection of pathogens using the 16S rRNA gene for bacteria and ITS region for fungi. This technology has demonstrated high sensitivity and specificity, thereby facilitating the establishment of targeted therapy. In this study, the authors developed this novel methodology by studying 53 hematopoietic stem cell transplant patients. Numerous infectious agents, including *A. versicolor*, were identified from blood samples. Meanwhile, Huang et al. [201] diagnosed invasive pulmonary aspergillosis from the blood samples of a patient with acute promyelocytic leukemia using NTS and identified *A. flavus* as the causative agent. In this case report, NTS technology was consistent with mNGS results; it is important to note that the diagnosis was made in 2 h, while the confirmatory result took 8 h. The implementation of this methodological technique contributed to patient improvement and the adjustment of antifungal treatment.

### 5.11. Isothermal Amplification Techniques Include Loop-Mediated Isothermal Amplification (LAMP)

This technique is characterized by requiring minimal equipment, its low cost, and representing an alternative to qPCR in low-resource settings [196]. Regarding assays for the identification of *Aspergillus* spp., efforts have been made, yet significant limitations have been evidenced, such as false positives in negative controls resulting from cross-contamination. Kobayashi et al. [202] conducted a study on 55 patients to evaluate the utility of LAMP in the diagnosis of CPA using sputum samples, identifying *A. fumigatus* and *A. flavus* as the primary etiological agents. This technique demonstrated a sensitivity and specificity of 55.9% (95% CI, 37.9–72.8%) and 100% (95% CI, 77.2–100.0%), respectively. It is worth mentioning that the subjectivity of the turbidimetric and colorimetric determination of the reaction depends on color perception. Consequently, other efforts have been made, such as the detection of *A. fumigatus* via LAMP coupled with a lateral flow biosensor. In a study by Jiang et al. [203], the authors reported 100% specificity with no cross-reactivity and results in less than 75 min, confirming this as a useful tool for the rapid and reliable identification of *A. fumigatus*.

### 5.12. Next-Generation Sequencing (NGS)

#### mNGS and tNGS

Although PCR has demonstrated great diagnostic utility, the limitations of its various variants, coupled with a lack of standardization, have driven new approaches that allow for pathogen detection even in cases of viral, bacterial, and fungal co-infections.

Next-Generation Sequencing is an emerging alternative in which all DNA fragments present in a sample are sequenced, followed by computational alignment that includes the referencing of the genomes present in the sample. As such, this process allows for the rapid detection, characterization, and genotyping of organisms that are difficult to culture, as well as the identification of virulence and resistance genes [110]. Another characteristic of NGS has been its diagnostic utility in the context of emerging fungal diseases, where endemicity may influence the failure to consider the possibility of various infectious agents being present [196]. Despite all the advantages of this technique, it must still be considered that clinical utility, as well as the standardization of this technique—particularly regarding interpretation—merits attention, and areas of opportunity have been identified.

Regarding specific populations at risk of developing aspergillosis, hematopoietic stem cell transplant patients have been among the most studied groups in this context. Particularly due to the variation in the diagnostic efficacy of tests such as GM and BDG, NGS has proven to be of diagnostic utility. Zhang et al. [204] evaluated the efficacy of mNGS vs. conventional microbiological tests. Their study included 46 patients with diagnoses of probable, proven, and undefined fungal infection. The mNGS technique allowed for the identification of *Aspergillus* spp. and reduced the time required for the identification of this fungal genus (*p* = 0.0016). Furthermore, it demonstrated greater sensitivity compared to conventional microbiological tests (*p* < 0.0001). Upon obtaining the identification results of the fungal agents, 55.26% of cases required modifications in the clinical approach, which translated into a post-transplant survival rate of 71.55% (95% CI, 55.18–85.82%).

It is important to mention that other efforts have sought to compare various methodologies, such as GM, BDG, and mNGS. In this sense, the efficacy of mNGS for the diagnosis of invasive pulmonary aspergillosis in patients with risk factors such as type 2 diabetes mellitus has been evaluated [205].

Liu et al. [205] included a total of 66 patients, of whom 21 presented with invasive pulmonary aspergillosis and the rest did not. In this study, mNGS demonstrated a sensitivity of 66.7% and a specificity of 100%, being superior to other methodologies, except for BAL culture, which had a sensitivity of 75.0%. However, the time to obtain results was superior for mNGS at 1.6 days. Additionally, mNGS detected co-infections, which allowed for the adjustment of treatment for these patients and thereby favored their outcomes.

Despite the significant advantages associated with the use of mNGS, important limitations remain and must be considered, such as the analysis of results to discriminate colonization from infection, which is related to the evaluation of the number of sequences, expertise in handling these technologies, and cost [193].

In addition to mNGS, another sequencing tool that has drawn attention in the clinical setting is targeted NGS (tNGS). This technique is based on multiplex PCR amplification or probe capture, which enriches the nucleic acids of known pathogens. Furthermore, it is characterized by being more accessible than mNGS while maintaining high sensitivity.

Chen et al. [206] compared the results of mNGS vs. tNGS in 61 patients with a diagnosis of invasive pulmonary fungal infection using BAL samples. The authors report that both methodologies showed a sensitivity of 95.08%, with a variation in specificity of 90.74% for mNGS and 85.19% for tNGS. The positive predictive value was 92.1% and 87.9%, with a negative predictive value of 94.2% and 93.9% for mNGS and tNGS, respectively. The most prevalent etiological agent identified by both methodologies was *A. fumigatus*. Meanwhile, Qin et al. [207] performed this same comparison in two groups of patients—immunocompromised and immunocompetent—with lower respiratory tract infections, without demonstrating statistical significance. Nonetheless, it was significantly higher compared to culture (*p* < 0.0001). Thus, existing evidence highlights the clinical validation of tNGS as being faster and less costly than mNGS.

### 5.13. Matrix-Assisted Laser Desorption–Ionization Time-of-Flight (MALDI-TOF)

Currently, another typing tool for *Aspergillus* spp. is MALDI-TOF. Due to its benefits, such as speed and precision in the identification of microorganisms, it has become one of the routine methodological tools in some laboratories, even in developing countries.

Despite the diagnostic complexity in identifying cryptic species of *Aspergillus* spp., MALDI-TOF appears to demonstrate excellent utility in differentiating between closely related species. Shao et al. [208] identified the limitations of this methodology using clinical and environmental samples. When comparing the Bruker library alone against the combined library (Bruker and supplementary database), the latter demonstrated superiority in identifying *Aspergillus* spp. strains (85.7% vs. 64.3%). Furthermore, the identification sensitivity was 100%, 86.5%, 76.1%, 100%, and 80% for strains from the *Fumigati*, *Flavi*, *Nigri*, *Terrei*, and *Nidulantes* sections, respectively. While it achieved a sensitivity of 71.4% in identifying other *Aspergillus* strains, some misidentifications among *A. niger*, *A. welwitschiae*, *A. luchuensis*, *A. flavus*, and *A. sydowii* were recognized. Despite the use of a library integrated by two different databases, the MALDI-TOF technique presented limited performance in distinguishing species from the *Nigri*, *Flavi*, and *Nidulantes* sections.

### 5.14. Volatile Organic Compounds (VOCs)

**Figure 3:**
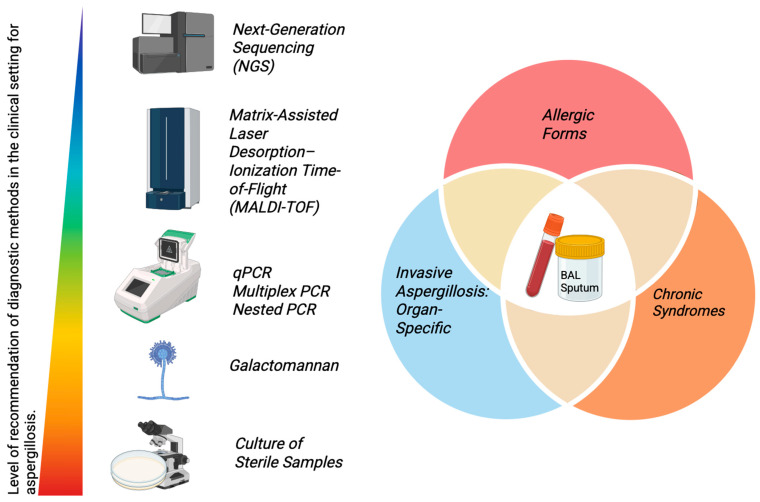
Main diagnostic tools for aspergillosis from clinical simples. Among the main diagnostic methods for the various clinical forms of aspergillosis, the culture of sterile samples such as bronchoalveolar lavage and blood stands out, followed by the identification of the cell wall component of *Aspergillus* spp. (galactomannan). Additionally, for confirmatory purposes and to evaluate antifungal resistance, PCR in its various forms (qPCR, multiplex PCR, and nested PCR) has been widely used. However, more sophisticated, albeit expensive, methodologies such as MALDI-TOF have been implemented in laboratories; however, it is not included in international guidelines, and its high cost limits its use. Meanwhile, more innovative methodologies such as next-generation sequencing are beginning to be used, although, like MALDI-TOF technology, it represents a high cost for laboratories, and due to its reliance on data, these results must be interpreted with caution. Created in Biorender. Carlos Alberto Castro-Fuentes. (2026) https://www.biorender.com.

The diagnostic tools described previously are performed by obtaining a patient sample (BAL, blood, sputum, etc.) (Figure 3). However, new diagnostic alternatives have been proposed for the diagnosis of aspergillosis. Belizario et al. [209] have proposed breath biopsies as an alternative for identifying infectious diseases based on the recognition of volatile organic compounds (VOCs) produced during an infection by an infectious agent. Specifically, for *A. fumigatus*, certain VOCs such as α-bergamotene and β-trans-bergamotene have been described as specific signatures for the diagnosis of pulmonary infection. Furthermore, Heddergott et al. [210] investigated the production of VOCs by *A. fumigatus* in vitro and evidenced that volatile production depended on the nutritional environment; they also demonstrated that *A. fumigatus* predominantly produces terpenes and related compounds. Recently, Li et al. [211] proposed a sensitive and specific assay for the diagnosis of chronic pulmonary aspergillosis. The authors included 53 CPA patients, 32 with community-acquired pneumonia, and 48 patients in the control group. Exhaled samples were analyzed using thermal desorption single-photon ionization time-of-flight mass spectrometry. In this regard, phenol, neopentyl alcohol, toluene, limonene, and ethylbenzene were identified, achieving a sensitivity of 95.8% and a specificity of 96.9%. Notably, the VOC limonene was positively correlated with anti-*A. fumigatus* IgG antibody titers (*p* < 0.01). Following treatment, both IgG levels and the VOC concentration decreased in the CPA patient group. Consequently, the utility of VOCs as biomarkers for differential diagnosis and the prediction of therapeutic response in patients with CPA was recognized. 

### 5.15. Artificial Intelligence (AI)

Recently, artificial intelligence has been making its way into the clinical context, contributing significantly through the training, validation, and testing of this promising and novel diagnostic tool. The proposal of artificial intelligence offers advantages over PCR and MALDI-TOF based on cost, as the latter have high expenses. Therefore, as an alternative, Tsang et al. [212] conducted a study on the automatic identification of clinically important *Aspergillus* species through image recognition. The authors performed the training stage using 6867 colonial images of *A. fumigatus*, *A. flavus*, *A. niger*, and *A. terreus*. Their results showed an accuracy of 99.35%, and the ResNet-18 neural network proved to be the most suitable algorithm. Furthermore, its speed, minimal need for specialized personnel, low reagent costs, and ease of use are promising. However, the main limitation is that it can only be carried out in cases where an *Aspergillus* spp. isolate has been obtained from the patient’s clinical sample.

## 6. Treatment

The therapeutic management of invasive pulmonary aspergillosis is fundamentally based on the identification of patient risk factors and the severity of the clinical presentation.

### 6.1. Voriconazole

Voriconazole is the first-line treatment for IPA, as it has demonstrated a significant reduction in mortality in both adult and pediatric populations. Early initiation of treatment, even in cases of possible IPA, is justified in patients with severe disease, even while the diagnostic evaluation is ongoing [9]. In this regard, the recommended standard dose in adults consists of a loading dose of 6 mg/kg intravenously every 12 h for the first day, followed by a maintenance dose of 4 mg/kg intravenously every 12 h, or its oral equivalent in clinically stable patients.

Due to the inhibition of multiple cytochrome P450 enzymes, voriconazole carries a high potential for drug–drug interactions, particularly in critically ill patients, with a reported incidence of up to 30% [213]. Among the most frequent adverse events is the elevation of transaminases; however, phototoxicity, skin reactions, neurotoxicity, and QTc interval prolongation have also been described [214]. While voriconazole-associated hepatotoxicity occurs most frequently during the first two weeks after starting treatment, it is usually reversible and generally resolves within approximately two weeks following dose adjustment or drug discontinuation [215].

In addition to adverse events, multiple factors contributing to the pharmacokinetic variability of voriconazole have been identified, including polymorphisms of the *CYP2C19* enzyme, age, liver function, and its non-linear pharmacokinetics. Since a correlation between serum concentrations and therapeutic efficacy has been demonstrated, therapeutic drug monitoring (TDM) of serum levels is recommended during administration [9].

Recent studies have shown that trough concentrations of voriconazole between 1.5 and 5.5 mcg/mL are adequate for the treatment of IPA [216]. Therefore, the IDSA recommends maintaining trough levels above 1–1.5 mcg/mL to ensure therapeutic efficacy, but below 5–6 mcg/mL to reduce the risk of neurotoxicity. In complicated, extensive, multifocal, disseminated infections, or those with central nervous system involvement, a higher trough target (>2 mcg/mL) is suggested [9]. Serum level measurement is typically performed between days 5 and 7 after treatment initiation or following dose adjustments, once steady state is reached. It should be mentioned that the recommendations for therapeutic ranges depend on the guide consulted, as well as the patient’s clinical context.

### 6.2. Isavuconazole

Recent updates to therapeutic guidelines include isavuconazole as a first-line treatment or as an alternative for invasive aspergillosis, particularly in scenarios where lower toxicity or better tolerability is sought.

The SECURE study, a phase III clinical trial, compared isavuconazole with voriconazole in patients with invasive aspergillosis, predominantly pulmonary. In that study, isavuconazole demonstrated non-inferiority to voriconazole in overall mortality at 42 days (18.6% vs. 20.2%), as well as similar rates of clinical and microbiological response, with a superior tolerability profile and a lower frequency of drug-attributable adverse events [217]. Beyond controlled clinical trials, various retrospective studies have confirmed the clinical efficacy and favorable safety profile of isavuconazole, even in high-risk populations such as patients with hematological malignancies [218,219].

From a pharmacokinetic perspective, isavuconazole does not routinely require therapeutic drug monitoring and presents a lower potential for cytochrome P450-mediated drug interactions compared to other azoles. Furthermore, it is associated with a lower frequency and severity of gastrointestinal adverse effects relative to voriconazole, resulting in a low rate of treatment discontinuation due to toxicity [220].

### 6.3. Amphotericin B

Amphotericin B deoxycholate and its lipid derivatives are an alternative treatment when azoles cannot be used or as salvage therapy. In patients with suspected azole resistance, it may be considered as a first-line treatment. Due to its safety profile, liposomal amphotericin B is preferred due to lower rates of nephrotoxicity and better tolerance than amphotericin B deoxycholate.

Emerging evidence indicates that nebulized liposomal amphotericin B is safe and well-tolerated; although direct therapeutic data in IPA are still preliminary, studies suggest favorable effects on biomarkers (galactomannan) and a decrease in inflammatory burden [221]. Additionally, other publications have documented intrabronchial treatment of fungal lesions with amphotericin B, achieving radiological and clinical improvement in IPA [222].

### 6.4. Echinocandins

Echinocandins are not recommended as monotherapy for IPA. However, they may be used as adjunctive treatment in salvage therapy.

In clinical practice, echinocandin monotherapy has shown inconsistent success rates, and there are currently no randomized clinical trials comparing its efficacy and safety against amphotericin B or triazoles.

### 6.5. Posaconazole

It has been described as an effective therapy for primary treatment and not only in rescue conditions; its favorable safety profile positions it especially for patients at risk of toxicity or with relevant drug–drug interactions.

A phase 3 clinical trial demonstrated non-inferiority compared to voriconazole in patients with proven or probable invasive aspergillosis; the overall clinical response was similar in both groups, and mortality rates were 25% for voriconazole vs. 21% for posaconazole, showing no statistically significant difference [223].

### 6.6. Refractory Disease

Refractory disease is defined as clinical worsening, the appearance of new signs or symptoms attributable to the infection, or the progression of radiological findings, despite the patient receiving antifungal treatment for at least two weeks [224]. In this regard, treatment for refractory disease must be individualized according to the extent of the disease, the patient’s severity, and comorbidities; it is also crucial to always rule out the presence of a new etiological agent. Therapeutic strategies described for refractory disease include switching the antifungal regimen, gradual reduction or suspension of immunosuppressive agents when possible, and surgical resection of necrotic lesions.

The use of combination therapy has been described in this scenario; however, there are no clinical trials demonstrating the superiority of this therapeutic strategy. The most frequently used drugs for salvage therapy are liposomal amphotericin B, posaconazole, micafungin, and anidulafungin. Combinations of echinocandins + triazoles or polyenes are the most clinically studied to date [225]. When using a new azole as salvage therapy, the possibility of potential antifungal resistance and previous exposure to antifungals must be considered.

### 6.7. Duration of Treatment

The duration of treatment is 6 to 12 weeks in most cases. In patients with persistent immunosuppression or extensive disease, therapy is maintained until radiological resolution and the disappearance of clinical signs, even beyond 12 weeks. This is because there is no absolute consensus on the exact duration; the decision must be adapted to the individual clinical context [9].

### 6.8. New Therapeutic Options

Due to the need for new therapeutic options for resistant aspergillosis, innovative molecules have recently been developed.

Olorofim represents the first-in-class antifungal of the orotomide family. Its mechanism of action consists of inhibiting dihydroorotate dehydrogenase, which blocks fungal pyrimidine synthesis and leads to cell death [226].

This drug has demonstrated fungicidal activity against azole-resistant *Aspergillus fumigatus* and has been used in cases of refractory disease. It is available in an oral formulation, with adequate bioavailability and good penetration into tissues such as the lungs and the central nervous system.

Clinical evidence comes from a phase 2 trial that included patients with invasive fungal disease lacking therapeutic options, including invasive pulmonary aspergillosis. In this study, an overall response rate of 28% was observed at 42 days of treatment, reaching up to 70% when considering stable disease. The most frequent adverse effects were gastrointestinal, with no deaths attributable to the treatment [227].

Fosmanogepix acts by inhibiting the *Gwt1* enzyme, which interferes with the synthesis of essential components of the fungal cell wall. It presents a broad spectrum of activity against *Aspergillus* spp., *Candida* spp., and mucorales and is available in both intravenous and oral formulations [228].

Clinical evidence has been documented in studies such as AEGIS [229], an open-label phase 2 clinical trial in which intravenous fosmanogepix was administered with subsequent transition to the oral formulation. Results showed a favorable clinical response in a significant proportion of patients, with encouraging survival rates given the severity of the studied population. Likewise, the drug demonstrated an adequate safety profile, with good tolerability and no relevant signals of toxicity.

Taken together, these findings position fosmanogepix as a promising therapeutic alternative in the management of refractory invasive fungal infections or those with limited treatment options.

### 6.9. Support and Follow-Up

In the management of IPA, supportive measures and follow-up are fundamental to optimize therapeutic response and reduce complications. Whenever clinically feasible, the reduction or adjustment of immunosuppression should be considered to favor the recovery of the host’s immune response. Radiological follow-up through serial computed tomography is essential to evaluate the progression or regression of pulmonary lesions and to guide the duration of treatment. Likewise, the use of biomarkers, such as serum galactomannan, can be useful for monitoring therapeutic response and carries prognostic value in neutropenic patients.

Finally, close monitoring of antifungal-associated toxicity is essential, with periodic checks of liver and renal function, as well as dosage adjustments based on therapeutic drug monitoring of serum levels when indicated.

### 6.10. Chronic Pulmonary Aspergillosis

Chronic pulmonary aspergillosis is a progressive entity that, in most cases, requires prolonged antifungal therapy, as well as a multidisciplinary approach involving specialists in infectious diseases, thoracic surgery, pulmonology, and radiology.

Oral azoles constitute the cornerstone of treatment, with itraconazole being the first-line option. It is administered for a minimum period of six months, although in many patients it may be extended depending on clinical evolution, radiological findings, and microbiological response [230].

According to the clinical presentation, indications for initiating pharmacological treatment include persistent symptoms such as productive cough for at least three months, progression of lung lesions on imaging studies, deterioration of the patient’s functional status, and hemoptysis.

The primary objective of treatment is to alleviate symptoms, prevent disease progression, and, in some cases, achieve a reduction in the size of pulmonary cavitations.

Due to the scarcity of clinical trials, the treatment of chronic aspergillosis lacks standardized guidelines and is primarily based on expert consensus (Table 2). In localized forms, such as simple aspergilloma, observation or surgery may be chosen, whereas more advanced or cavitary disease requires sustained antifungal treatment.

## 7. Resistance in Aspergillosis

Resistance surveillance of *Aspergillus* spp. is not a routine strategy compared to other microorganisms, given its low global incidence. Specific measurements in Denmark between 2018 and 2020 reported a 3.6% resistance rate in clinical isolates of *A. fumigatus* [232]. A multicenter study involving 29 hospitals in Spain reported 7.4% [233], while in Europe, the range oscillates between 1% and 11% [234]. These figures should be interpreted with caution, as it is uncommon to have the proper equipment for fungal identification, and the volume of cases limits species-specific segregation.

The clinical scenario in which a resistant *Aspergillus* spp. infection is expected, as previously mentioned, includes patients with sustained primary and secondary immunodeficiency and prior exposure to azole treatment. *A. fumigatus* resistance to this type of therapy is widely documented, and its management represents a clinical challenge [235,236].

The primary resistance mechanisms include tandem repeats in the *cyp51A* promoter and coding substitutions, with TR34/L98H and TR46/Y121F/T289A being among the most frequent. Additionally, the overexpression of efflux pumps via ABC (abcG1) and MFS (mdr1/mfs) transporters, as well as mutations in the ergosterol synthesis pathway, contribute synergistically to the resistant phenotype [236,237].

Another particularly relevant aspect is the selection of resistant *A. fumigatus* strains favored by environmental exposure to agricultural azoles. This environmental pressure is associated with the use of Demethylation Inhibitors (DMIs) employed in cereal crops such as wheat, barley, and maize, as well as fruits (apple, pear, citrus), other vegetables, and ornamental horticulture [237,238]. In this context, azole residues in plant waste and agricultural soils create environmental niches that allow for the selection of genotypes carrying characteristic *cyp51A* mutations [237].

Other fungicides used, such as strobilurins (soybean and maize crops) and benzimidazoles (rice, wheat, and vegetables), although not directly inducing cross-resistance to human-use azoles, contribute to the selection of environmental *Aspergillus* spp. strains. It is pertinent to underscore that these azole fungicides are not directed toward treating *Aspergillus* spp. but toward controlling phytopathogenic fungi, primarily Ascomycetes (*Erysiphe*, *Blumeria*, *Fusarium graminearum*) and some Basidiomycetes (*Puccinia*, *Uromyces*, *Rhizoctonia*) [238].

Current evidence is still insufficient to quantitatively assess the impact of these fungicides on the increase in human cases of resistant aspergillosis. However, some studies have documented the retrieval of clinically relevant isolates with resistance genotypes acquired in the environment, even from patients without prior exposure to human-use azoles [237,239,240]. These findings justify the need to address the issue from a One Health perspective, integrating environmental and clinical surveillance [235,240].

Furthermore, the persistence of the microorganism and the difficulty of the immune system in its clearance circumscribe the clinical scenarios conducive to the development of resistance. This may be due to either a failed immune response or the microorganism’s own evasion mechanisms [241,242].

In *A. fumigatus* specifically, specific mechanisms allowing its persistence in the host have been described. The presence of melanin in its conidia interferes with phagosome maturation and contributes to complement inactivation [242]. Likewise, it has been described that molecules such as C3 and C5 can be inactivated by fungal proteases like Mep1p and Alp1p [243,244,245]. Additionally, galactosaminogalactan secreted by fungal hyphae allows for the masking of cell wall β-glucan and is associated with reduced recognition and the induction of neutrophil apoptosis [241,246]. Also, the production of immunosuppressive toxins such as gliotoxin contributes by reducing macrophage phagocytosis and favoring their apoptosis [4,241,242,247]. Finally, the formation of biofilms or structures such as aspergillomas hinders access for immune cells, allowing the microorganism to persist post-treatment and fostering a scenario for the development of resistance [241,248].

In Table 3 summarizes the main resistance mechanisms according to the treatments described in the previous section.

## 8. Conclusions

This review highlights the global importance of aspergillosis, as the data provided in this work demonstrate that this mycosis is recorded worldwide. Furthermore, it reveals that although *A. fumigatus* is the main etiological agent, other causal species exist and must be considered, which demands constant epidemiological and clinical monitoring.

It is also important to emphasize the new risk factors for immunocompetent populations described in this review and to consider the continuous spectrum of clinical forms of aspergillosis, in addition to considering populations with underlying pulmonary disorders such as chronic obstructive pulmonary disease and interstitial lung diseases.

PCR in its various forms, MALDI-TOF, and NGS were the main methodologies used for diagnosing aspergillosis.

Furthermore, the effectiveness of isavuconazole compared to voriconazole in treating aspergillosis was demonstrated. While novel antifungals such as olorofim and fosmanogepix show excellent results in the management of aspergillosis, it is important to consider antifungal resistance acquired through exposure to prevent overuse.

Furthermore, the data included in this work strengthen the need to individualize aspergillosis cases for improved management and antifungal treatment.

## Figures and Tables

**Table 1:** Risk factors reported during the information search period for the development of aspergillosis.

Risk Factor	Odds Ratio (OR)	Lower Limit (95% CI)	Upper Limit (95% CI)	*p*-Value	Reference
*Hematological patient*					
Cytomegalovirus infection or reactivation	2.57	0.76	8.73	NA	[82]
*Cancer patient*					
Low albumin level (27.00, CI: 20.00–33.90 g/L)	0.80	0.27	1.88	0.002	[160]
Respiratory failure	12.7	10.2	15.2	0.005	
Febrile neutropenia	7.33	5.21	9.45	0.007	
Sex (male)	1.96	NA	NA	0.008	[161]
Past and present smoker	2.92	NA	NA	<0.001	
Chronic obstructive pulmonary disease	1.88	NA	NA	0.011	
Interstitial lung disease	3.71	NA	NA	0.001	
Pulmonary tuberculosis	2.79	NA	NA	0.028	
Treatment with double lobectomy	2.74	NA	NA	<0.001	
*Transplant p* *atient*					
Post-transplant kidney replacement	3.36	1.78	6.34	NA	[162]
Post-transplant cytomegalovirus disease	2.81	1.47	5.36	NA	
Chronic lung diseases	7.26	1.05	50.06	NA	[83]
Diabetic nephropathy	1.65	1.10	2.48	NA	
Post-transplant hemodialysis	3.69	2.13	6.37	NA	
Surgical reintervention	6.28	1.67	23.66	NA	
Post-transplant bacterial infection	7.51	4.37	12.91	NA	
Viral respiratory tract infection	7.75	1.60	37.57	NA	
Cytomegalovirus disease or infection	2.67	1.12	6.32	NA	
Acute graft rejection	3.01	1.78	5.09	NA	
Delayed renal graft function	10.60	1.05	106.84	0.045	[163]
Use of systemic antibiotic	4.74	NA	NA	0.03	[164]
History of pneumonia	48.7	NA	NA	0.01	
*Other pathologies*					
*Interstitial lung disease patient*					
Lymphopenia	2.74	1.34	5.60	<0.05	[165]
Honeycomb	2.91	1.42	5.94	<0.05	
*Non-neutropenic patients*					
Use of glucocorticoids	30.22	2.67	341.30	NA	[166]
Admission to the intensive care unit	0.13	0.02	0.75	NA	
Arterial partial pressure of oxygen/inspired fraction of oxygen ratio PaO_2_/FiO_2_) ratio (187.18 ± 94.97 vs. 264.15 ± 97.47)	0.99	0.99	0.99	NA	
*Allergic bronchopulmonary aspergillosis patient*					
Sex (female)	2.44	1.15	5.16	0.020	[167]
Specific IgE for *A. fumigatus* 10.10 kUA/L (3.38, 23.50)	1.05	1.02	1.08	0.002	
Presence of bronchiectasis	3.61	1.07	12.21	0.039	
*Coinfections in patient*					
*Fungus*					
*Pneumocystis jirovecii*					
Lactic acidosis	33.99	3.11	371.40	0.004	[168]
Low CD4+ count (<114 cells/µL)	19.34	1.53	259.38	0.022	
High LDH level (> 519 U/L)	11.42	1.27	102.66	0.030	
*Virus*					
*Influenza*					
Solid organ transplant	4.8	1.7	13.8	NA	[169]
Hematological malignancy	2.5	1.5	4.1	NA	
Immunocompromise	2.2	1.6	3.1	NA	
Prolonged corticosteroid treatment prior to hospital admission	2.4	1.4	4.3	NA	
Liver cirrhosis	6.7	2.1	19.4	<0.01	[170]
Hematological malignancy	3.3	1.2	8.5	0.02	
Influenza A(H1N1)pdm09 subtype	3.9	1.6	9.1	<0.01	
Vasopressor requirement	4.1	1.6	12.7	<0.01	
History of smoking during the last year	6.2	1.7	26	NA	[171]
The use of antibiotics for more than 7 days prior to admission	4.89	1.0	89	NA	
*Non-influenza*					
Cumulative dose of prednisone > 140 mg within the first 7 days	22.6	4.5	112	NA	[172]
Pneumonia at the time of acute non-infectious respiratory infection	7.2	1.6	31.7	NA	
*SARS-CoV-2*					
High-dose corticosteroids for 7 days (>420 mg/week)	1.73	0.35	8.57	NA	[173]
Prolonged use of corticosteroids	2.79	0.63	13.92	NA	
Body mass index (23.2 ± 4.7 kg/m^2^, mean)	1.27	1.08	1.50	0.01	[174]
Solid organ malignancy	5.37	1.35	21.33	0.02	
Age (>62 years)	2.34	1.39	3.92	0.001	[175]
Use of dexamethasone and anti-IL-6	2.71	1.12	6.56	0.027	
Prolonged mechanical ventilation (>14 days)	2.16	1.14	4.09	0.019	
Chronic liver disease	2.70	1.21	6.04	0.02	[92]
Hematological malignancy	2.47	1.27	4.83	0.008	
Chronic obstructive pulmonary disease	2.00	1.42	2.83	<0.000	
Cerebrovascular disease	1.31	1.01	1.71	0.059	
Mechanical ventilation	2.83	1.88	4.24	<0.000	
Kidney transplant therapy	2.26	1.76	2.90	<0.000	
Treatment of COVID-19 with IL-6	2.88	1.52	5.43	0.001	
Corticosteroid treatment	1.88	1.28	2.77	0.001	
Steroid dose > 60 mg of dexamethasone	3.77	1.03	13.79	NA	[176]
Chronic lung disease	4.20	1.26	14.02	NA	
Treatment with azithromycin for 3 days or more	3.1	1.1	8.5	0.02	[177]
Fever	3.3	1.01	11.09	0.048	[178]
Lymphocytopenia	4.3	1.2	14.7	0.019	
Unvaccinated patients	6.6	1.7	25.1	0.006	
*Phlebovirus*					
CD4+ count < 68 cells/mm^3^ in conjunction with a CD8+ count < 111 cells/mm^3^	0.21	0.05	0.80	0.022	[179]
>99 pg/mL IL-6 combined with >111 pg/mL IL-10	17.61	2.31	133.76	0.006	
*Hospital setting*					
Positive result for Galactomannan	3.1	1.2	8.0	0.021	[180]
Pulmonary reactivation due to cytomegalovirus during intubation	5.3	1.1	26.8	0.043	
Isolation of *Aspergillus fumigatus*	1.9	0.8	4.6	NA	[181]
Isolation of *Aspergillus nidulans*	1	0.08	12	NA	
Isolation of *Aspergillus niger*	1.2	0.25	5.7	NA	
Liposomal amphotericin B	1.04	0.3	3.55	NA	
Combination of amphotericin B liposomes with posaconazole	3.33	0.28	38.7	NA	
Age (40 years old, median)	1.03	1.01	1.05	NA	
Sex	1.1	0.47	2.56	NA	
Stay in ICU	4.27	1.73	10.53	NA	
Hematological malignancy	2.48	1.07	5.73	NA	
CKD	3.67	1.6	8.5	NA	
Prolonged use of corticosteroids	1.56	0.7	3.48	NA	
Mechanical ventilation	2.77	1.21	6.36	NA	
Sepsis	3.67	1.15	11.72	NA	
High galactomannan antigen index (value of ≥1)	1.6	0.72	3.56	NA	
Chronic lung disease	4.12	1.72	9.84	0.001	[182]
Intermittent positive pressure ventilation rate	3.15	1.09	9.07	0.033	
Irreversible acute kidney injury	13.36	4.52	39.48	<0.001	
Corticosteroid use within one year	2.89	1.20	6.95	0.018	
*Pediatric patients*					
Sex (male)	2.45	NA	NA	NA	[183]
Atopic dermatitis	3.15	NA	NA	NA	
Sensitivity to another fungal genus (*Alternaria* spp.)	10.37	NA	NA	NA	
Longer duration of asthma	1.26	NA	NA	NA	
IL-8 level (0.5 (0.3 to 0.7) log scale)	0.5	0.3	0.7	<0.001	[184]
Neutrophil count percentage (8.2 (4.8 to 11.6) log scale)	8.2	4.8	11.6	<0.001	
Neutrophil elastase level (0.5 (0.3 to 0.7) log scale)	0.5	0.3	0.7	<0.001	
Number of admissions for intravenous antibiotic therapy 0.2 (0.1 to 0.3)	0.2	0.1	03	0.008	

LDH: Lactate Dehydrogenase; ICU: Intensive care unit; CKD: Chronic Kidney Disease; NA: Not available.

**Table 2:** Recommended treatment regimens for the management of chronic pulmonary aspergillosis.

Clinical Form	First-Line Treatment	Treatment Alternatives	Treatment Duration	Reference
Simple aspergilloma	Observation in asymptomatic patients.	Surgery for significant hemoptysis Consider peri- and postoperative antifungal treatment if there is a risk of rupture. If surgery is contraindicated, administer prolonged azole therapy. Instill amphotericin B into the aspergilloma cavity via bronchoscopy or percutaneously. In cases of hemoptysis, consider administration of tranexamic acid and arterial embolization.	Individualized	[112,230,231]
Chronic cavitary pulmonary aspergillosis	Itraconazole	Voriconazole/posaconazole; switch due to toxicity or resistance	≥6–12 months (frequent prolonged)	[112]
Chronic fibrosing aspergillosis	Itraconazole or voriconazole	Posaconazole; multi-disciplinary management for the treatment of pulmonary fibrosis	Long term (years)	[112]

**Table 3:** Molecular Mechanisms of Antifungal Resistance in *Aspergillus* spp.

Antifungal Group	Antifungal	Mutations That Most Significantly Impair Its Function	Key Insight	Reference
Triazole (14-α-demethylase (*CYP51A*) Inhibition)	Voriconazole	Tandem repeat: TR34/L98H, TR46/Y121F/T289A. Point mutations: G138C, Y431C	Tandem mutations specifically impair voriconazole, whereas point mutations trigger cross-resistance across the spectrum	[236,249]
	Isavuconazole	Point mutation: G54(E/W/R/K), G138C	G54- impacts posaconazole more significantly than voriconazole whereas	[250]
	Posaconazole	Point mutation: G54(E/W/R/K), G138C	G138C confers a broad cross-resistance spectrum	
Echinocandins (β-1,3-D-glucan synthases (FKS1)inhibition)	Micafungin	FKS1: point mutation: E671Q. Tolerance through cell wall remodeling. Biofilm matrix remodeling	FKS1 mutations are deleterious across all echinocandins.	[232,251]
	Anidulafungin		Increased cell wall chitin is an expected stress response.	
	Caspofungin		Alanine metabolism–dependent biofilm matrix remodeling	
Polyene (Ergosterol binding–dependent membrane pore formation)	Amphotericin B	Ergosterol biosynthesis disruption	Reduced ergosterol diminishes amphotericin B binding. No recurrent clinical hot spot mutation described in *Aspergillus*	[250]

## Data Availability

No new data were created or analyzed in this study. Data sharing is not applicable to this article.

## Supplementary Materials

The following supporting information can be downloaded at: https://www.mdpi.com/article/10.3390/jof12030229/s1, Table S1: Species of the genus *Aspergillus*: Geographic origin, origin of samples and methods of species identification. References [13,14,15,16,17,18,19,20,21,22,23,24,25,26,27,28,29,30,31,32,33,34,35,36,37,38,39,40,41,42,43,44,45,46,47,48,49,50,51,52,53,54,55,56,57,58,59,60,61,62,63,64,65,66,67,68,69,70,71,72,73,74,75,76,77,78,79] are cited in the supplementary materials.
