# Supplementary material for: Aspergillosis: An Update on Epidemiology, Risk Factors, Diagnosis, Susceptibility, and Treatment
Source: J Fungi (Basel). 2026 Mar 21;12(3):229. doi: 10.3390/jof12030229 (PMC13027440; doi:10.3390/jof12030229)
Supplement: Supplementary file 1 [file jof-12-00229-s001.zip › jof-4129394-supplementary.pdf]

Table S1. Species of the genus *Aspergillus*: Geographic origin, origin of samples and methods of species identification.

Reference	Country of Study	Origin of the samples (number of samples)	Identification Method(s)	Species Identified (number of isolates)
[13]	Peru	Clinical samples:<br>Respiratory (116)<br>Skin and annexes (17)<br>Miscellaneous (10)	Colony morphology and microscopic morphology<br>Sequencing *benA* gene and ITS region	Colony morphology and microscopic morphology:<br>*Aspergillus fumigatus* and *A. flavus* (37)<br><br>Sequencing *benA* gene and ITS region:<br>*A. fumigatus sensu stricto* (144)
[14]	France	Clinical samples:<br>Respiratory samples (68)<br>External auditory canal (34)<br>Ear, nose, and throat sphere (5)	Sequencing *benA* gene	*A. tubingensis* (51)<br>*A. welwitschiae* (50)<br>*A. niger* (7)<br>*A. neoniger* (2)<br>*A. japonicus* (1)<br>*A. brasiliensis* (1)
[15]	Argentina	Clinical samples:<br>Sputum (50)	Colony morphology and microscopic morphology<br>Sequencing *benA* and *CaM* genes and ITS region	*Aspergillus* section *Fumigati*:<br>*A. fumigatus* (76)<br>*A. lentulus* (3)<br><br>*Aspergillus* section *Flavi*<br>*A. flavus* (18)<br>*A. parasiticus* (1)<br>*A. pseudonomius* (1)<br><br>*Aspergillus* section *Terrei*<br>*A. terreus* (14)<br>*A. pseudoterreus* (1)<br>*A. alabamensis* (1)<br><br>*Aspergillus* section *Nigri*<br>*A. niger* (8)<br><br>*Aspergillus* section *Usti*<br>*A. calidoustus* (1)

				Aspergillus section *Nidulante*<br>*A. nidulans* (1)
[16]	France and Denmar k	Clinical samples:<br>Respiratory tract (47)<br>Sinus fungal ball (1)<br>Environmental sample (2)	Sequencing *benA* and *CaM* genes	*Aspergillus* section *Fumigati:*<br>*A. thermomutatus* (18)<br>*A. hiratsukae* (12)<br>*A. lentulus* (9)<br>*A. felis* (4)<br>*A. udagawae* (4)<br>*A. fischeri* (3)<br>*A. tsurutae* (1)
[17]	Taiwan and Mainlan d China	Clinical samples:<br>Sputum (166)<br>BAL (64)<br>Nasal or throat swabs (22)<br>Tracheal aspirates (11)<br>Wound discharge (93)<br>Effusion or drainage (12)<br>Others (22)	Sequencing ITS, *benA* and *CaM* genes	*Aspergillus* section *Fumigati*:<br>*A. fumigatus* (108)<br>*A. lentulus* (1)<br>*A. turcosus* (1)<br><br>*Aspergillus* section *Flavi*:<br>*A. flavus* (93)<br>*A. nomius* (4)<br>*A. tamarii* (3)<br>*A. oryzae* (1)<br><br>*Aspergillus* section *Nigri*:<br>*A. niger* (75)<br>*A. tubingensis* (7)<br>*A. japonicus* (5)<br>*A. aculeatus* (2)<br>*A. luchuensis* (1)<br>*A. cristatus* (1)<br><br>*Aspergillus* section *Terrei*:<br>*A. terreus* (1)<br><br>*Aspergilus* section *Nidulantes*:<br>*A. nidulans* (11)<br>*A. unguis* (1)<br>*A. quadrlineatu* (1)<br>*A. delacrixii* (1)

				Aspergillus section *Versicolores:*<br>*A. versicolor* (14)<br>*A. sydowii* (11)<br>*A. austroafricanus* (1)<br><br>*Aspergillus* section *Usti*:<br>*A. ustus* (1)<br>*A. inseutus* (1)<br><br>*Aspergillus* section *Circumdati*:<br>*A. caesiellus* (1)<br><br>*Aspergillus* section *Aspergillus*:<br>*A. pseudoglaucus* (1)
[18]	Iran	Clinical samples:<br>BAL (34)<br>Biopsy (6)<br>Tracheal secretions (5)	Colony morphology and microscopic morphology<br>Sequencing *benA* gene	*A. terreus* (33)<br>*A. nidalans* (7)<br>*A. latus* (2)<br>*A. ochraceus* (1)<br>*A. citrinoterreus* (1)
[19]	Pakistan	Clinical samples:<br>IA (25)<br>CPA (89)<br>ABPA (58)<br>Severe asthma (4)<br>Saprophytic tracheobronchial aspergillosis (23)	Colony morphology and microscopic morphology	*A. fumigatus* (38)<br>*A. flavus* (64)<br>*A. niger* (9)<br>*A. terreus* (3)
[20]	Japan	Clinical samples (44):<br>Sputum<br>BAL<br>Endotracheal aspirate<br>Surgical samples from the lower respiratory tract	Colony morphology and microscopic morphology<br>Sequencing *benA* gene	*Aspergillus* section *Nigri*:<br>*A. welwitschiae* (22)<br>*A. tubingensis* (17)<br>*A. niger* (4)<br>*A. uvarum* (1)
[21]	Japan	Clinical samples:<br>Sputum samples (45)<br>Otorrhea samples (27)<br>Bronchial aspirates (14)	Sequencing *benA, CaM* genes and ITS region and/or hydrophobin (rodA) were determined	*A. fumigatus* (67)<br>*A. welwitschiae* (10)<br>*A. tubingensis* (8)<br>*A. flavus* (7)

		Wounds samples (10)<br>BAL (3)<br>Pleural effusion samples (3)<br>Fecal samples (3)<br>Bronchial brushing (2)<br>Drain samples (2)<br>Pus samples (2)<br>Pharyngeal mucus sample (1)<br>Urine sample (1)<br>Nasal discharge sample (1)<br>Lung abscess sample (1)<br>Postnasal cavity sample (1)<br>Tonsil sample (1)<br>Respiratory sample (1)<br>Uknown source sample (1)<br>.	to identify the species in *Aspergillus* section *Fumigati*<br>Sequencing *benA* gene were used to identify species in *Aspergillus* sections *Flavi, Nigri,* and *Nidulantes*	*A nidulans* (6)<br>*A. terreus* (6)<br>*A. niger* (3)
[22]	Hong Kong and China	Clinical samples (160)	Colony and microscopic morphology<br>Sequencing *benA* and *CaM* genes and ITS region	ITS sequencing (126):<br>*A. fumigatus* (83)<br>*A. flavus* (25)<br>*A. terreus* (11)<br>*A. niger* (7)<br><br>Analysis of concatenated ITS, *benA* and *CaM* sequences (34):<br><br>*Aspergillus* section *Flavi*:<br>*A. tamarii* (4)<br>A. *pseudocaelatus* (3)<br>*A. pseudonomiae* (1)<br><br>*Aspergillus* section *Nidulantes*:<br>*A. amoenus* (2)<br>*A. sydowii* (2)<br>*A. austroafricanus* (1)<br>*A. sublatus* (1)<br>*A. nidulans* (1)<br>*A. tabacinus* (1)<br><br>*Aspergillus* section *Nigri*:<br>*A. welwitschiae* (7)<br>*A. tubingensis* (5)<br>*A. brunneoviolaceus* (4)<br>*A. costaricensis* (1)

				Aspergilus section *Restricti*:<br>*A. restrictus* (1)
[23]	China	Clinical samples:<br>BAL (98)	Colony morphology and microscopic morphology<br>Sequencing *benA* and *CaM* genes and ITS region	*A. fumigatus* (57)<br>*A. flavus* (23)<br>*A. niger* (15)<br>*A. sydowii* (1)<br>*A. terreus* (1)<br>*A. nidulans* (1)
[24]	China	Samples obtained of patients with otomycosis (45)	Morphology in combination with BLAST analysis of the generated *benA* sequences.	Phenotypically<br>*Aspergillus* section *Nigri* (26)<br>*Aspergillus* section *Fumigati* (8),<br>*Aspergillus* section *Terrei* (7)<br>*Aspergillus* section *Flavi* (4)<br><br>Sequencing *benA* gene<br>*A. tubingensis* (18)<br>*A. niger* (6)<br>*A. welwitschiae* (2)<br>*A. fumigatus* (8)<br>*A. terreus* (7)<br>*A. flavus* (4)
[25]	Iran	Clinical samples:<br>Bronchoalveolar lavage (29)<br>Endotracheal (5)<br>Sputum (4)<br>Sinus discharge (3)<br>Lung biopsy (1)<br>Ear swabs (1)<br>Environmental samples (23)	Colony morphology and microscopic morphology<br>Sequencing *benA* gene	*A .fumigatus*
[26]	Austria	Clinical samples:<br>BAL(18)<br>Sputum (21)<br>Ear (11)	Sequencing *CaM* gene	Clinical samples:<br>*A. terreus* (50)<br>*A. floccosus* (1)

		Wound swabs (1)<br>Soil samples (3485)		Soil samples:<br>*A. fumigatus* (2398)<br>*A. flavus* (889)<br>*A. niger* (887)<br>*A terreus* (189)
[27]	Switzerland	Clinical samples:<br>Lung (24)<br>Skin/soft tissue (4)<br>Brain (33)<br>Bone (2)<br>Other (1)	Sequencing *benA and CaM* genes	*A. calidoustus* (19)<br>*A. pseudodeflectus* (2)<br>*A. ustus sensu sricto* (1)<br>*A. insuetus* (2)<br>*A. keveii* (1)<br>*A. puniceus* (1)
[28]	Iran	Clinical samples:<br>BAL (71)<br>Pulmonary and respiratory disorders (33)<br>Solid organ transplantation (19)<br>Invasive fungal rhinosinusitis (14)<br>Haematological malignancies (12)<br>Injury (7)<br>Autoimmune diseases and corticosteroid consumption (6)<br>Sinusitis (5)<br>Bone marrow transplantation (4)	Macroscopic and microscopic morphology<br>Sequencing *CaM* gene	*A. flavus* (95)<br>*A. tubingensis* (23)<br>*A. welwitschiae* (18)<br>*A. fumigatus* (13)<br>*A. sydowii* (6)<br>*A. neoniger* (2)<br>*A. citrinoterreus* (2)<br>*A. terreus* (2)<br>*A. tritici* (1)<br>*A. ochraceus* (1)<br>*A. nidulans* (1)<br>*A. montevidensis* (1)<br>*A. minisclerotigenes* (1)<br>*A. rugulosus* (1)<br>*A. tabacinus* (1)<br>*A. ustus* (1)<br>*A. niger* (1)<br>*A. japonicus* (1)
[29]	Líbano	Clinical samples:<br>Ears (60)<br>Lower respiratory (8)<br>Nails (5)	Macroscopic and microscopic morphology<br>MALDI-TOF MS<br>Sequencing *BenA* and *CaM* genes	*A. niger (40)*<br>*A. flavus* (20)<br>*A. tubingensis* (4)<br>*A. fumigatus* (3)<br>*A. terreus* (3)<br>*A. sydowy* (1)<br>*A. welwitschiae* (1)<br>*A. tamarii* (1)
[30]	Italia	Clinical samples:<br>Respiratory samples (403)<br>Auricular secretions (6)	Macroscopic and microscopic morphology	*Aspergillus* section *Fumigati:*<br>*A. fumigatus* (286)

		Skin/wound (6)<br>Biopsies (4)<br>Cerebral abscess (3)<br>Corneal scraping (3)	to identification level section<br>Sequencing *benA* gene were used for the molecular identification of *Aspergillus* section *Nigri* isolates	*A. thermomutatus* (1)<br><br>*Aspergillus* section *Flavi:*<br>*A. flavus* (61)<br><br>*Aspergillus* section Nigri**:**<br>*A. niger* (42)<br><br>*Aspergillus* section *Terrei:*<br>*A. terreus* (29)<br><br>*Aspergillus* section *Nidulantes***:**<br>*A nidulans* (2)<br><br>*Aspergillus* section *Versicolores*:<br>*A. versicolor* (2)<br><br>*Aspergillus* section *Aspergillus*:<br>*A. amstelodami* (1)<br><br>*Aspergillus* section *Usti*:<br>*A. insuetus* (1)
[31]	México	Clinical samples:<br>BAL (47)<br>Sputum (46)<br>Tracheal aspirate (18)<br>Nasal tissue (9)<br>Others (9)	Macroscopic and microscopic morphology to identification level section<br>Sequencing *benA* and *CaM* genes and ITS region	Sequencing ITS region:<br>*Aspergillus* section *Fumigati* (47)<br>*Aspergillus* section *Flavi* (37)<br>*Aspergillus* section *Nigri* (33)<br>*Aspergillus* section *Terrei* (19)<br>*Aspergillus* section *Versicolores* (5)<br>*Aspergillus* section *Usti* (2))<br>*Aspergillus* section *Nidulantes (1)*<br>*Aspergillus* section *Circumdati* (1)<br>*Aspergillus* section *Clavati* (1)

				Sequencing *benA* and *CaM* genes:<br>*Aspergillus* section *Nigri (17):*<br>*A. tubingensis (13)*<br>*A. aculeatus (3)*<br>*A. aculeatinus (1)*<br><br>*Aspergillus* section *Flavi (6):*<br>*A. tamari (5)*<br>*A. nomius (1)*<br><br>*Aspergillus* section *Fumigati* (5)<br>*A. fumisynnematus* (4)<br>*A. hiratsukae* (1)<br><br>*Aspergillus* section *Versicolores* (5):<br>*A. sydowii* (4)<br>*A. griseoaurantiacus* (1)<br><br>*Aspergillus* section *Usti* (2):<br>*A. calidoustus* (2)<br><br>*Aspergillus* section *Nidulantes* (1)<br>*A. rugosus* (1)<br><br>*Aspergillus* section *Circumdati* (1)<br>*A. ochraceus* (1)
[32]	USA	Clinical samples:<br>Lower respiratory tract (896)<br>Upper respiratory tract (853)<br>Eyes/orbit (79)<br>Tissue not otherwise specified (77)<br>Extrapulmonary fluid (52)<br>Extremities (45)<br>CNS (43)	Macroscopic and microscopic morphology<br>Sequencing *benA* and *CaM* genes	*A. fumigatus* (2072)<br>*A. lentulus* (29)<br>*A. hiratsukae* (13)<br>*A. thermomutatus* (8)<br>*A. udagawae* (6)<br>*A. fumigatiaffinis* (4)<br>*A. fumisynnematus*(2)<br>*A. fischeri* (1)<br>*A. nishimurae* (1)

		Abscess fluid drainage (33)<br>Abdomen/gastrointestinal tract (22)<br>Cardiovascular (15)<br>Blood/urine (15)<br>Bone (8)		*A. pseudoviridinutans* (1)<br>*A. viridinutans* (1)
[33]	Iran	Clinical samples:<br>BAL (68)<br>Sputum (34)<br>Ear (48)<br>Eye (6)<br>Heart biopsy (9)<br>Sinuses biopsy (24)<br>Abscess (2)<br>Environment samples (46)	*Aspergillus* isolates were identified at the species complex level by their colonial morphology. Species-level identification was performed by PCR-RFLP and sequencing the *benA* gene	Colony and microscopic morphology:<br>*Aspergillus* section *Flavi* (117)<br>*Aspergillus* section *Nigri* (77)<br>*Aspergillus* section *Fumigati* (21)<br>*Aspergillus* section *Terrei* (14)<br>*A. pseudodeflectus* (2)<br>*A. melleus* (2)<br><br>PCR-RFLP and sequencing *benA* gene:<br>*A. pseudodeflectus* (1)<br>*A. niger* (16)<br>*A. melleus* (1)<br>*A. flavus* (2)<br>*A. luchuensis* (5)<br>*A. tubingensis* (23)
[34]	Serbia	Clinical samples:<br>Ear samples (30)	Colony and microscopic morphology	*A. niger* complex (20)<br>*A. flavus* complex (10)
[35]	Switzerl and	Clinical samples:<br>Tissue	Direct examination, colony and microscopic morphology and panfungal PCR<br>Sequencing *benA* gene	Direct examination, colony and microscopic morphology and panfungal PCR:<br>*Aspergillus* section *Nigri*<br><br>Sequencing *benA* gene:<br>*A. tubingensis*
[36]	Spain	Clinical samples (109)	Colony and microscopic morphology<br>Sequencing *CaM* gene	Morphological identification:<br>*A. fumigatus* (76)

				A. flavus (10),<br>*A. niger* (6)<br>*A. terreus* (5)<br>Cryptic species (10)<br><br>Sequencing *CaM* gene:<br>*A. alliaceus* (1)<br>*A. montevidensis* (1)<br>*A. arcoverdensis* (2)<br>*A. lentulus* (2)<br>*A. tubingensis* (1)<br>*A. ellipticus* (2)<br>*A. nomius* (1)
[37]	China	Clinical samples:<br>Ear swab (69)	*Aspergillus* isolates were identified at the species complex level by their colonial morphology. Species-level identification was performed by MALDI-TOF MS	Colonial morphology**:**<br>*Aspergillus* section *Nigri* (48)<br>*Aspergillus* section *Terrei* (11)<br>*Aspergillus* section *Flavi* (8)<br>*Aspergillus* section *Fumigati* (2)<br><br>MALDI-TOF MS:<br>*A. welwitschiae* (25)<br>*A. niger* (11)<br>*A. tubingensis* (12)<br>*A. terreus* (11)<br>*A. flavus/A. oryzae* (8)<br>*A. fumigatus* (2)
[38]	Iran	Clinical samples:<br>Ears (88)<br>Nails (42)<br>Sputum (83)	Sequencing *benA* gene	*A. flavus* (110)<br>*A. pseudonomius* (1)<br>*A. alliaceus* (1)<br>*A. minisclerotigenes* (1)<br>*A. niger* (41)<br>*A. tubingensis* (49)<br>*A. welwtschiae* (5)<br>*A. luchuensis/awamori* (3)<br>*A. japonicus* (2)

[39]	Switzerland	Clinical samples:<br>Sputum (237)<br>Tracheobronchial aspirate (94)<br>BAL (38)<br>Upper respiratory samples (18)<br>Bronchial/lung biopsy (13)	MALDI-TOF MS and/or sequencing *benA* and *CaM* genes	*A. fumigatus* complex (355)<br>*A. niger* complex (20)<br>*A. flavus* complex (12)
[40]	Indonesia	Clinical samples:<br>Sputum (59)	Colony and microscopic morphology<br>Sequencing *benA* and *CaM* genes and ITS region	*Aspergillus* section *Fumigati*:<br>*A. fumigatus* (28)<br><br>*Aspergullus* section *Flavi*<br>*A. flavus* (7)<br><br>*Aspergillus* section *Nigri*<br>*A. niger* (7)<br>*A. welwitschiae* (1)<br>*A. tubingensis* (3)<br>*A. brunneoviolaceum* (7)<br>*A. aculeatus* (2)<br>*A. neoniger* (2)<br><br>*Aspergillus* section *Clavati*<br>*A. clavatus* (1)<br>*A. tamarii* (1)
[41]	Turkey	Clinical samples:<br>BAL (20)<br>Tissue (10)<br>Wounds (5)<br>Sterile body fluids (5)<br>Sputum (4)	Colony and microscopic morphology<br>Sequencing ITS region	*A. fumigatus* (23)<br>*A. niger* (12)<br>*A. flavus* (6)<br>*A. terreus* (3)
[42]	Japan	Clinical samples (126):	Direct examination<br>Colony and microscopic morphology<br>DNA sequencing of the D1/D2 and ITS regions, *benA*,<br>and *CaM* genes	*Aspergillus* section *Fumigati* (86)<br>*A. fumigatus sensu stricto* (85)<br>*A. lentulus* (1)<br><br>*Aspergillus* section *Nigri* (31)<br>*A. niger sensu stricto* (1)<br>*A. tubingensis* (18)<br>*A. welwitschiae* (11)

				A. luchuensis (1)<br><br>*Aspergillus* section *Flavi* (6)<br>*A. flavus sensu stricto* (6)<br><br>*Aspergillus* section *Terrei* (3)<br>*A. terreus* (1)
[43]	Japan	Clinical samples:<br>Sputum (1)<br>Environment samples (4)	MALDI-TOF MS<br>Sequencing *benA*, *CaM*, McM7, *RPB2*, *Tsr1* genes and ITS region	MALDI-TOF MS:<br>*A. niger* (5)<br><br>Sequencing *CaM* gene:<br>*A. tubingensis* (4)<br>*A. welwitschiae* (1)<br><br>Sequencing ITS region:<br>*A. tubingensis* (4)<br>NA (1)<br><br>Sequencing *benA* gene:<br>*A. tubingensis* (4)<br>NA (1)<br><br>Sequencing Mcm7 gene:<br>*A. tubingensis* (4)<br>NA (1)<br><br>Sequencing RPB2 gene:<br>*A. tubingensis* (4)<br>NA (1)<br><br>Sequencing Tsr1 gene:<br>*A. tubingensis* (4)<br>NA (1)
[44]	China	Clinical samples:<br>Sputum (123)<br>BALF (12)<br>Sterile sites (20)<br>Colorectal Content (1)	Macroscopic and microscopic morphology and MALDI-TOF MS<br>Sequencing ITS region	*A. fumigatus* (88)<br>*A. flavus* (32)<br>*A: niger* (24)<br>*A. versicolor* (3)<br>*A. terreus* (3)<br>*A. nidulans* (1)

				Others (5)
[45]	Japan	Clinical samples:<br>Brain biopsy (3)<br>Pleural fluid (1)<br>Autopsy (1)<br>TBLB (3)<br>Sinus biopsy (1)<br>VATS (1)	Sequencing of internal transcribed spacer region, the D1/D2 region of the rRNA gene, and *benA* gene	*A. turcosus* (1)<br>*A. felis* (1)<br>*A. viridinutans* (1)<br>*A. nidulans* (1)<br>*A. calidoustus* (1)<br>*A. fumigatus* (3)<br>NA (2)
[46]	Kenya	Sputum (25)	Colony morphology and microscopic morphology was done<br>ELISA to detect *A. fumigatus* IgG antibodies	*A. fumigatus* (13)<br>*A. niger* (8)<br>*A. terreus* (1)<br>*A. flavus* (1)<br>*A. candidus* (1)<br>*A. clavatus* (1)
[47]	Ghana	Sputum (38)	Colony morphology and microscopic morphology<br>LD- Bio *Aspergillus* IgG and IgM LFA	*A. fumigatus* (18)<br>*A. niger* (14)<br>*A. flavus* (5)<br>*A. terreus* (1)
[48]	China	Clinical samples:<br>Sputum (294)<br>BALF (101)<br>Ear secretion (41)<br>Pus (29)	Colony and microscopic morphology<br>MALDI-TOF MS	*Aspergillus* section *Fumigati* (357)<br>*Aspergillus* section *Nigri* (47)<br>*Aspergillus* section *Flavi* (42)<br>*Aspergillus* section *Terrei* (28)
[49]	USA	Clinical samples:<br>Lower respiratory tract (130)<br>Upper respiratory tract (63)<br>Lower extremity (20)<br>Upper extremity (8)<br>Back/abdomen (8)<br>Head (7)<br>Blood (5)<br>Eye (5)<br>Lung tissue (5)<br>Heart (4)<br>Bone (3)<br>Unknown (20)	Colony and microscopic morphology<br>Sequencing *benA, CaM* and *RPB2* genes and ITS region	Sequencing ITS region:<br>*A. heldtiae*<br>*A. alabamensis*<br>*A. terreus*<br>*A. hortae*<br><br>Sequencing ***benA*** gene:<br>*A. alabamensis*<br>*A. jilinensis*<br>*A. heldtiae*<br>*A. hortae*<br><br>Sequencing *CaM* gene:

				A. heldtiae<br>*A. jilinensis*<br>*A. alabamensis*<br>*A. citrinoterreus*<br><br>Sequencing *RPB2* gene*:*<br>*A. alabamensis*<br>*A. heldtiae*<br>*A. citrinoterreus*<br>*A. hortae*
[50]	Iran	Clinical samples:<br>Otomycosis patients (134)	Colony and microscopic morphology<br>Sequencing *CaM* gene	Colony and microscopic morphology*:*<br>*Aspergillus* section *Nigri* (83)<br>*Aspergillus* section *Flavi* (35)<br>*Aspergillus* section *Terrei* (1)<br>*Aspergillus* section *Fumigati* (83)<br><br>Sequencing *CaM* gene:<br>*A. welwitschiae* (52)<br>*A. tubingensis* (31)<br>*A. niger*(2)<br>*A. neoniger* (1)
[51]	France	Clinical samples:<br>Rrespiratory tract (33)<br>Toenail onychomycosis (5)<br>Otomycosis (1)<br>Respiratory samples (2)<br>Nail (1)<br>Environment samples (6)	Sequencing *benA* and *CaM*<br>genes	*A. sclerotiorum* (17),<br>*A. persii* (5)<br>*A. subramanianii* (1)<br>*A. westerdijkiae* (13)<br>*A. ochraceus* (5)<br>*A. affinis* (1)<br>*A. ostianus* (1)<br>*A. insulicola* (5)<br>*A. ochraceopetaliformis* (4)
[52]	France	Clinical samples:<br>Cancer treatment center (3)<br>Skin scales of right foot (2)<br>Sputum (2)<br>Nail of big toe (2)	Sequencing *benA* gene	*A. amoenus* (1)<br>*A. creber* (10)<br>*A. fructus* (1)<br>*A. jensenii* (4)<br>*A. protuberus* (1)

		Scalp (1)<br>BALF (11)<br>Armpit skin (1)<br>External auditory canal (1)<br>Environment samples (7)		*A. puulaauensis* (1)<br>*A. sydowii* (11)<br>*A. tabacinus* (1)
[53]	Korea	Clinical samples:<br>BAL (31)	Colony and microscopic morphology<br>Sequencing *benA* gene	*Aspergillus* section *Nigri* (15):<br>*Aspergillus* section *Fumigati* (13)<br>*Aspergillus* section *Flavi* (2)<br>*Aspergillus* section *Terrei* (1)
[54]	China	Clinical samples (491):<br>Respiratory tract<br>BALF<br>Sputum<br>Corneal scrapings<br>Ear canal secretions	Colony and microscopic morphology and MALDI-TOF MS<br>Sequencing *benA* gene and ITS region	*A. fumigatus sensu lato* (252)<br>*A. flavus* (169)<br>*A. terreus* (37)<br>*A. niger* (29)<br>*A. nidulans* (4)<br>*A. neoellipticus* (2)
[55]	Spain	Clinical samples (283)<br>Environmental samples (52)	Colony and microscopic morphology TRESPERG typing	Clinical/Environmental**:**<br>*A. fumigatus* (139/35)<br>*A. niger* (40/10)<br>*A. flavus* (27/1)<br>*A. nidulans* (15/3)<br>*A. lentulus* (11/0)<br>*A. calidoustus* (3/0)<br>*A. carneus* (1/0)<br>*A. unguis* (3/2)<br>*A. candidus* (2/0)<br>*A. versicolor* (3/0)<br>*A. fumigatiaffinis* (1/0)<br>*N. udagawae* (3/0)<br>*A. ochraceus* (3/0)<br>*A. sydowii* (1/0)
[56]	Turkey	Clinical samples:<br>Respiratory tract samples (45)	Colony and microscopic morphology<br>Sequencing ITS region	Colony and microscopic morphology:<br>*A. fumigatus* (20)

				A. terreus (3)<br>*A. flavus* (6)<br>*A. niger* (3)<br>*Aspergillus* spp. (3)<br><br>Sequencing ITS region:<br>*A. fumigatus* (20)<br>*A. terreus* (3)<br>*A. flavus* (6)<br>*A. niger* (4)<br>*A. welwitschiae* (2)
[57]	Iran	Clinical samples:<br>BAL (101)<br>Tracheal secretion (34)<br>Pleural fluid (6)<br>Chest tube (4)<br>Biopsy (3)<br>Wound (1)	Colony and microscopic morphology<br>Sequencing *benA* gene	Colony and microscopic morphology:<br>*A. flavus* (101)<br>*A. fumigatus* (49)<br><br>Sequencing *benA* gene:<br>*A. flavus* (101)<br>*A. fumigatus* (49)
[58]	Australia	Clinical samples:<br>Tissue (81)<br>BAL (65)<br>Upper respiratory tract specimens (47)<br>Intra-operative or deep swab (26)<br>Sterile site fluid (17)	Sequencing ITS region	*Aspergillus* section *Fumigati* (119)<br>*Aspergillus* section *Flavi* (36)<br>*A. terreus* (32)<br>*A. niger* (29)<br><br>Other uncommon species identified (n = 20):<br>*A. nidulans*<br>*A. calidoustus*<br>*A. sydowii*<br>*A. ochraceus*<br>*A. viridinutans*<br>*A. insuetus*<br>*A. unguis*<br>*A. ustus*<br>*A. wisconsinensis*<br>*A. lentulus*
[59]	Taiwan	Clinical samples:<br>Sputum (241)	Colony and microscopic morphology	*Aspergillus* section *Fumigati* (119):

		BAL (48)<br>Pleural fluid (3)<br>Lung tissue (2)<br>Ear (65)<br>Unspecified pus (53)<br>Skin including hair and nail (30)<br>Unspecified tissue (18)<br>Nasal cavity/paranasal sinus (6)<br>Eye and cornea (5)<br>Others (21)	Sequencing *CaM* gene	*A. fumigatus* (118)<br>*A. thermomutatus* (1)<br><br>*Aspergillus* section *Flavi* (165):<br>*A. flavus* (158)<br>*A. pseudonomius* (4)<br>*A. tamarii* (2)<br>*A. nomius* (1)<br><br>*Aspergillus* section *Nigri* (128):<br>*A. welwitschiae* (77)<br>*A. niger* (16)<br>*A. brunneoviolaceus* (14)<br>*A. tubingensis* (13)<br>*A. neoniger* (4)<br>*A. aculeatinus* (1)<br>*A. costaricaensis* (1)<br>*A. japonicus* (1)<br>*A luchuensis* (1)<br><br>*Aspergillus* section *Terrei* (49):<br>*A. terreus* (49)<br><br>*Aspergillus* section *Nidulantes:*<br>*A. unguis* (3)<br>*A. nidulans* (2)<br><br>Series *Versicolores* (20):<br>*A. sydowii* (10)<br>*A. austroafricanus* (4)<br>*A. tabacinus* (2)<br>*A. fructus* (1)<br>*A. protuberus* (1)<br>*A. versicolor* (1)<br>ser. *Versicolores* (1)<br><br>*Aspergillus* section *Circumdati* (4):<br>*A. flocsosus* (1)<br>*A. occultus* (1)<br>*A. subramanianii* (1)<br>*A. westerdijkiae* (1)

				Aspergillus section *Restricti* (1):<br>*A. restrictus* (1)<br><br>*Aspergillus* section *Aspergillus* (1):<br>*A. chavalieri* (1)
[60]	Chile	Clinical samples:<br>BALF (23)	Colony and microscopic morphology<br>Sequencing *benA* gene and ITS1-5.8S-ITS2 region	Colony and microscopic morphology:<br>*Aspergillus* section *Fumigati* (23)<br><br>Sequencing *benA* gene and ITS1-5.8S-ITS2 region:<br>*A. fumigatus sensu stricto* (23)
[61]	Iran	Clinical samples:<br>Sputum (120)	Colony and microscopic morphology<br>Sequencing *benA* gene	*Aspergillus* section *Fumigati* (40)<br>*Aspergillus* section *Flavi* (36)<br>*Aspergillus* section *Nigri* (6)<br>*Aspergillus* section *Terrei* (4)
[62]	Italy	Clinical samples :<br>Sputum (521)<br>BAL (448)<br>Wound/biopsy (57)<br>Ear swab (39)<br>Nasal swab (42)<br>Sinus discharge (3)<br>Pleural fluid (14)<br>CSF (1)<br>Eye swab (1)	Colony and microscopic morphology<br>MALDI-TOF MS	*A. fumigatus* (497)<br>*A. niger* (251)<br>*A. flavus* (199)<br>*A. terreus* (119)<br>Others (60):<br>*A. nidulans*<br>*A. candidus*<br>*A. sclerotiorum*<br>*A. versicolor*<br>*A. lentulus*<br>*A. glaucus*<br>*A. usutus*<br>*A. oryzae*<br>*A. clavatus*<br>*A. ochraceus*

[63]	Denmar k	*A. flavus* obtained of clinical samples (140):<br>Sputum<br>Tracheal suction<br>Feces<br>BAL<br>Biopsy<br>Spinal fluid<br><br>Environmental samples (258)	Microsatellite typing	Clinical samples<br>*A. flavus* (140)<br><br>Environmental samples:<br>*A. fumigatus* (17)<br>*A. flavus* (9)<br>*A. calidoustus* (9)<br>*A. niger* (6)<br>*A. sydowii* (2)
[64]	Iran	Hospital environmental samples (693)	Sequencing *benA* gene	*Aspergillus* section *Fumigati*:<br>*A. fumigatus* (40)<br><br>*Aspergillus* section *Flavi*<br>*A. flavus* (5)<br>*A. oryzae* (4)<br><br>*Aspergillus* section *Nigri*<br>*A. tubingensis* (21)<br>*A. niger* (14)<br>*A. welwitschiae* (2)<br>*A. luchuensis* (2)<br>*A. japonicus* (2)<br>*A. awamori* (1)<br><br>*Aspergillus* section *Circumdati*<br>*A. terreus* (1)
[65]	China	Clinical samples (276):<br>Sputum (246)<br>BAL (27)<br>Wounds (2)	Colony and microscopic morphology<br>MALDI-TOF MS<br>Sequencing *benA* and *CaM* genes	Colony and microscopic morphology and MALDI-TOF MS:<br>*A. fumigatus* (276)<br><br>Sequencing *benA* and *CaM* genes:<br>*A. fumigatus* (276)
[66]	China	Clinical samples (3558):<br>BAL<br>Biopsy<br>Sputum	Colony and microscopic morphology<br>PCR<br>MALDI-TOF MS	*A. fumigatus* (2679)<br>*A. flavus* (437)<br>*A. niger* (219)<br>*A. terreus* (119)

		Bronchoscopy<br>Others (NA)	VITEK 2 COMPACT<br>Others (NA)	*A. nidulans* (35)<br>*A. tubingensis* (21)<br>*A. sydowii* (18)
[67]	Brazil	Samples of indoor air of critical areas of a hospital (324)	Colony and microscopic morphology<br>Sequencing *benA* and *CaM* genes	*Aspergillus* section *Fumigati* (95)<br>*Aspergillus* section *Nidulantes* (90)<br>*Aspergillus* section *Nigri* (89)<br>*Aspergillus* section *Flavi* (38)<br>*Aspergillus* section *Terrei* (10)
[68]	Spain	Clinical sample:<br>Tissue (1)	Colony and microscopic morphology<br>Sequencing ITS region and ribosomal large subunit D1-D2	Colony and microscopic morphology:<br>*Aspergillus*<br><br>Sequencing ITS region and ribosomal large subunit D1-D2:<br>*A. hiratsukae*
[69]	China	Clinical samples:<br>Ears canal secretions (452)	Colony and microscopic morphology<br>MALDI-TOF MS<br>Sequencing ITS region	*A. terreus* (284)<br>*A. flavus* (92)<br>*A. niger* (55)
[70]	Netherlands and Spain	Clinical samples (155), previously morphologically and/or molecularly identified as *A. sydowii*	Sequencing *CaM* gene	*A. sydowii* (145)<br>*A. versicolor (3)*<br>*A. creber (7)*
[71]	Malaysia	Clinical samples (28):<br>Sputum<br>BAL<br>Dermatology specimens.	Colony and microscopic morphology<br>Sequencing *benA* gene and ITS region	*A. niger* (17)<br>*A. fumigatus* (6)<br>*A. flavus* (3)<br>*A. chavalieri* (1)<br>*A. tubingensis* (1)

[72]	Ghana	Histopathology (21)	Histopathology: Periodic Acid-Schiff (PAS)<br>Grocott-Gomori Methenamine Silver (GMS)	*Aspergillus* sp. (21)
[73]	Brazil	Clinical sample:<br>BAL (1)	Colony and microscopic morphology<br>MALDI-TOF MS<br>Sequencing *benA* gene	Colony and microscopic morphology:<br>*Aspergillus* section *Nigri*<br><br>MALDI-TOF MS:<br>*A. welwitschiae*<br><br>Sequencing *benA* gene<br>*A. welwitschiae*
[74]	China	Clinical samples (120):<br>External auditory canal<br>Cornea<br>Subungual debris	Colony and microscopic morphology<br>MALDI-TOF MS<br>Sequencing *benA, CaM, RPB2* genes and ITS region	MALDI-TOF MS:<br>ser. *Terrei* (28)<br>ser. *Fumigati* (5)<br>ser. *Flavi* (5)<br>*Aspergillus* spp .(15)<br>Non-*Aspergillus* (7)<br>None (55)<br><br>Sequencing *benA, CaM, RPB2* genes and ITS region:<br>ser. *Nigri:*<br>*A. welwitschiae* (19)<br>*A. tubingensis* (3)<br>*A. niger* (2)<br>*A. vadensis* (1)<br><br>ser. *Flavi:*<br>*A. flavus/A. oryzae (10)*<br>*A. sojae (2)*<br><br>ser. *Fumigati:*<br>*A. fumigatus* (6)<br><br>ser. *Versicolores*<br>*A. versicolor* (1)<br>*A. sydowii* (1)<br><br>ser. *Terrei* :

				A. terreus (70)<br>*A. hortae (4)*
[75]	Iran	Clinical samples:<br>BAL (18)<br>Sputum (2)<br>Otomycosis lesions (98)<br>Environmental samples:<br>Soil (50)<br>Air (24)	Colony and microscopic morphology<br>Sequencing *Cam* gene	Clinical samples:<br>*A. tubingensis* (41)<br>*A. welwitschiae* (52)<br>*A. niger* (20)<br>*A. neoniger* (1)<br>*A. piperis* (2)<br>*A. luchuensis* (2)<br><br>Environmental samples:<br>*A. tubingensis* (38)<br>*A. welwitschiae* (17)<br>*A. niger* (14)<br>*A. neoniger* (2)<br>*A. piperis* (1)<br>*A. aculeatus* (2)
[76]	Iran	Clinical samples (127):<br>BAL (NA)<br>Endotracheal tube secretions (NA)<br>Sputum (NA)<br>Nasal biopsy (NA)	Colony and microscopic morphology<br>Sequencing *CaM* gene	*A. flavus sensu stricto* (93)<br>*A. fumigatus sensu stricto* (17)<br>*A. terreus sensu stricto* (7)<br>*A. niger sensu stricto* (6)<br>*A. candidus* (1)<br>*A. citrinoterreus* (1)<br>*A. tubingensis* (1)<br>*A. fumigatiaffinis* (1)
[77]	China	Clinical samples:<br>Ear canal secretions (62)<br>Sputum (45)<br>BAL (2)<br>Other body fluid secretions (2)	Colony and microscopic morphology<br>Sequencing *benA* gene and *ITS* region	*A. fumigatus* (45)<br>*A. niger* (20)<br>*A. tubingensis* (18)<br>*A. terreus* (15)<br>*A. flavus* (7)<br>*A. welwitschiae* (3)<br>*A. fumigatiaffinis* (2)<br>*A. lentulus* (1)
[78]	China	Clinical samples (95):<br>Sputum (NA)<br>BAL (NA)<br>Ear canal secretions (NA)<br>Tissue(NA)	Colony and microscopic morphology<br>MALDI-TOF MS	*A. fumigatus (54)*<br>*A. niger (19)*<br>*A. tubingensis (9)*<br>*A. flavus (10)*<br>*A. terreus (4)*

			Sequencing *benA* and *CaM,* genes and ITS1-5.8S-ITS2 region	*A. tamarii* (2)<br>*A. usamil* (1)<br>*A. versicolor* (1)<br>*A. udagawae* (1)<br>*A. lentulus* (1)<br>*A. sydowii* (1)<br>*A. quadrilineatus* (1).
[79]	Nigeria	Sputum samples (81)	Colony morphology and microscopic morphology	*A. fumigatus* (51)<br>*A. flavus* (16)<br>*A. niger* (14)

ben A: β-tubulin
ITS: Internal Transcribed Spacer
CaM: calmodulin
BAL: Bronchoalveolar Lavage
IA: Invasive Aspergillosis
CPA: Chronic Pulmonary Aspergillosis
ABPA: Allergic Bronchopulmonary Aspergillosis
BLAST: Basic Local Alignment Search Tool
MALDI TOF: Matrix-Assisted Laser Desorption–Ionization Time-of-Flight
CNS: Central Nervous System
PCR RFLP: Polymerase Chain Reaction-Restriction Fragment Length Polymorphism (PCR-RFLP)
D1/D2: Region of the Large Subunit of the 28S rDNA
RPB2: RNA polymerase II subunit B
Tsr1: Ribosome Biogenesis Factor Loci
TBLB: Transbronchial Lung Biopsy
VATS: Video-Assisted Thoracic Surgery
LFA: Lateral Flow Assay
TRESPERG: Novel Genotyping Method Based on Hypervariable TRs Within Exons of Surface Protein Coding Genes (TRESP) and A Fourth Target, erg4B Gene (Afu1g07140).
CSF: Cerebro Spinal Fluid
NA: Not available
